# Theoretical Modeling of Magnetoactive Elastomers on Different Scales: A State-of-the-Art Review

**DOI:** 10.3390/polym14194096

**Published:** 2022-09-29

**Authors:** Timur A. Nadzharyan, Mikhail Shamonin, Elena Yu. Kramarenko

**Affiliations:** 1 Faculty of Physics, Lomonosov Moscow State University, 119991 Moscow, Russia; 2 East Bavarian Centre for Intelligent Materials (EBACIM), Ostbayerische Technische Hochschule (OTH) Regensburg, Seybothstr. 2, 93053 Regensburg, Germany; 3 A.N. Nesmeyanov Institute of Organoelement Compounds, Russian Academy of Sciences (INEOS RAS), 119991 Moscow, Russia; 4 Enikilopov Institute of Synthetic Polymeric Materials, Russian Academy of Sciences (ISPM RAS), 117393 Moscow, Russia

**Keywords:** magnetoactive elastomers, magnetorheological elastomers, theoretical modeling, magnetic particles, mechanical properties, magnetorheological effect, magnetodeformation

## Abstract

A review of the latest theoretical advances in the description of magnetomechanical effects and phenomena observed in magnetoactive elastomers (MAEs), i.e., polymer networks filled with magnetic micro- and/or nanoparticles, under the action of external magnetic fields is presented. Theoretical modeling of magnetomechanical coupling is considered on various spatial scales: from the behavior of individual magnetic particles constrained in an elastic medium to the mechanical properties of an MAE sample as a whole. It is demonstrated how theoretical models enable qualitative and quantitative interpretation of experimental results. The limitations and challenges of current approaches are discussed and some information about the most promising lines of research in this area is provided. The review is aimed at specialists involved in the study of not only the magnetomechanical properties of MAEs, but also a wide range of other physical phenomena occurring in magnetic polymer composites in external magnetic fields.

## 1. Introduction

Magnetoactive elastomers (MAEs) are composite materials consisting of micro- or nanometer-sized magnetic particles embedded into a complaint elastomeric matrix [1,2,3,4,5,6,7,8,9,10]. They belong to the class of smart (or intelligent) materials because their physical properties or macroscopic response can be significantly changed in a controlled fashion by the application of moderate (a few hundred mT) magnetic fields [1,8,9]. Specifically, these are mechanical properties (e.g., the static and dynamic Young’s and shear moduli) and different electromagnetic properties (e.g., magnetization reversal curves, magnetic permeability, electrical conductivity and dielectric permittivity) [8,9]. The most prominent effect is the magnetorheological (MR) effect, which is a significant change of the shear storage and loss moduli in external magnetic fields. Due to this, MAEs are also known as magnetorheological elastomers [4]. Furthermore, MAE samples show pronounced deformations both in uniform and non-uniform external magnetic fields. If an MAE sample is placed in a uniform magnetic field, the corresponding changes in its shape or dimensions are usually referred to as magnetostriction, although the physical mechanism is different from that of magnetostriction in conventional solid magnetic materials [8,9]. In non-uniform magnetic fields, one speaks about the magnetodeformation of MAE samples, which can reach 200–300% [9]. Application of a uniform magnetic field also causes huge changes in dielectric properties of MAEs, particularly a relative increase in the effective dielectric permittivity reaches 1000% in moderate magnetic fields up to 0.6 T [9,11]. A detailed description of the wide range of magneto-responsive properties of MAEs can be found in recent reviews [8,9].

The main physical reason for all these effects is believed to be the restructuring of the ferromagnetic filler particles. This is their mutual re-arrangement in external magnetic fields (a change in their relative positions or, equivalently, change in the microstructure of a composite material) [8]. This argument is analogous to the effect observed in MR fluids where particles rearrange along the magnetic field lines forming elongated aggregates. A noticeable re-arrangement is only possible if the polymeric matrix is soft with the shear modulus below 100 kPa [9].

The interest in MAEs is determined by their prospective applications as active vibration absorbers, vibration isolators for mechanical engineering applications, base isolators for civil engineering applications, sensors and actuators [2,4,12,13,14,15,16]. Magnetically controlled dielectric and electric properties of MAEs open an opportunity to use MAEs as sensors of magnetic fields, as well as to consider them as tunable dielectrics [17,18], which find numerous applications as tunable filters, phase shifters, passive microwave components, or in phased array antenna, etc. Hitherto the majority of fundamental and applied research on MAEs was focused on utilization of bulk properties of these materials. However, it has been recently understood that MAEs are very promising materials for rapid and reversible control of various surface properties, in particular wettability [19,20,21,22], surface roughness [20,23], adhesion [24,25], and friction [26]. It opens up new opportunities for applications of MAE-based smart surfaces in various areas, e.g., droplet-based microfluidics, liquid transporters/distributors, fog harvesters and soft-robot locomotion.

**Figure 1:**
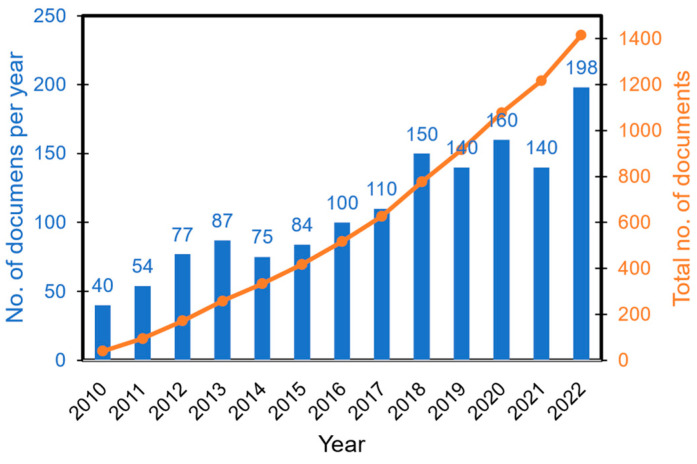
The number of published documents per year and the total number of documents since 2010 according to Google Scholar. The search is done according to the following terms in the title: “magnetoactive elastomer” OR “magnetoactive elastomers” OR “magnetoactive polymer” OR “magnetoactive polymers” OR “magnetorheological elastomer” OR “magnetorheological elastomers”. The results for the year 2022 are linearly extrapolated from the available data on 15 September 2022.

The field of MAE studies is developing rapidly. According to Google Scholar search the total amount of papers published in this field since 2010 would exceed 1400 at the end of 2022 (Figure 1). Several comprehensive reviews are available that focus on fabrication, characterization and applications of these materials [2,4,5,27,28]. As far as the theoretical description of the behavior of MAEs in external magnetic field is concerned, the latest review is about 6 years old [7], although several aspects of theoretical modeling have been also discussed in recent papers [28,29]. We believe that enough notable works have been published in the last five years to warrant an up-to-date review of advancements in theoretical modeling of MAEs. It is worth noting that we do not pretend to compile all the published theoretical works in the field of MAEs, but rather to overview the actual development in the field. Existing trends and those lines of research which would benefit greatly from increased activity in the future are identified. The focus of this review is on the theoretical description of the relationships between the external magnetic field and the resulting mechanical properties (elastic moduli, viscoelastic properties) and phenomena (magnetostriction and magnetodeformation). These effects are referred to as magnetomechanical coupling. This is the field where the majority of published theoretical works is concentrated. Obviously, the reason for that lies in the most promising application area of MAEs. Additional highly interesting physical effects (magnetic properties, magneto-electric effect, magnetoconductivity, surface properties, etc.) are mentioned in the framework of utilized approaches for the description of magneto-mechanical coupling but are not considered in detail. The theoretical works on non-mechanical and surface properties of MAEs deserve a separate review paper.

The paper is organized as follows: In Section 2 the underlying mechanisms that cause magnetomechanical coupling in MAEs are analyzed. In Section 3 the main approaches for modeling of MAEs are presented. Proposed classification of these approaches is based on the concept of different spatial scales that are utilized for modeling these composites. The (combined) multi-scale theoretical approaches are considered in Section 4. Advantages and disadvantages of the existing theoretical methods are discussed and the most promising lines of research in each section are identified. The results are summarized in the concluding section.

## 2. Basic Mechanisms behind Magneto-Mechanical Coupling

Restructuring of the ferromagnetic filler is commonly accepted as the underlying physical phenomenon for the majority of the effects discussed in Section 1, however a unified theoretical approach suitable for describing and predicting the wide spectrum of characteristics and responses of mechanically soft MAEs has not been developed yet. This can be attributed to the large variability in the material composition and the necessity to take into account nonlinear properties of constitutive materials. For example, the ferromagnetic particles can be either soft magnetic (e.g., carbonyl iron) or hard magnetic (e.g., NdFeB), and they can have different shapes, e.g., spherical or flake-like. Furthermore, the MAE samples can be cross-linked either in the absence of a magnetic field, which results in almost isotropic distribution of magnetizable filling particles, or in an external DC magnetic field, which creates anisotropic filler particle distribution. The magnetization of ferromagnetic particles demonstrates nonlinear dependence on the internal magnetic field, and, for hard magnetic particles, the magnetic hysteresis can’t be neglected. When ferromagnetic particles are displaced (translated and/or rotated) in an applied magnetic field, the surrounding polymeric matrix is deformed. It should be noted that the matrix can be chemically attached (grafted) to the particles via the functional particle/matrix interface or be physically adsorbed on the particle surface. As a result, magnetic interactions (both between individual magnetized particles as well as between each particle and the external magnetic field) and elastic forces arising due to matrix deformations compete when a magnetic field is applied to an MAE specimen. In general, elastomer matrices exhibit nonlinear viscoelastic behavior, which further complicates theoretical description. It is obvious that a large variety of synthesis conditions, material compositions, specimen shapes and excitation conditions (magnitude, direction and temporal behavior of an external magnetic field) leads to the need for a comprehensive multi-scale model for MAE materials.

**Figure 2:**
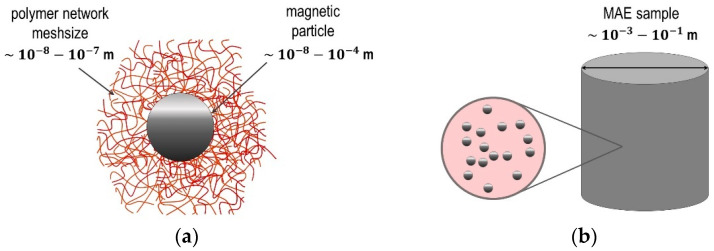
Schematic representation of MAE’s multiscale structure: (**a**) a magnetic particle in a polymer matrix resolved at the nanoscale; (**b**) a macroscopic MAE sample with a random distribution of magnetic particles in a viscoelastic medium.

Figure 2 schematically shows different scales which should be addressed in the theoretical description of MAE composites. These scales are defined here as follows: microscopic (polymer network, multidomain magnetic structure of µm-sized particles, etc.), mesoscopic (granularity and filler particles as separate physical objects) and macroscopic (larger than the correlation length, specimen scale). It should be mentioned that the mesh size of the polymer network can be comparable to the particle diameter only in the case of magnetic nanoparticles. Typically, the particles are larger than the length of network subchains (Figure 2a). This scale difference can reach several orders of magnitude in the case of µm-sized particles which are commonly used as MAE filler, so that the approach in which the polymer matrix is viewed as a continuum medium (Figure 2b, magnifying glass) is fully justified. At the macroscopic scale (Figure 2b), the MAE sample can be considered as a continuum medium with given magnetic and elastic characteristics.

Inherent complexity and nonlinearity of the fully coupled magnetomechanical problem call for various approximations, which will be discussed in the following sections. An obvious simplification is to provide theoretical description for a particular spatial scale. Therefore, it was decided to classify the theoretical approaches to modeling of MAEs on the basis of the scale considered in each work.

## 3. Main MAE Modeling Approaches

### 3.1. Microscopic and Mesoscopic Modeling

Modeling the microscopic structure of the material and its evolution in the magnetic field is the most fundamental approach to MAE behavior description. In so-called “bottom-up” models, local behavior of individual particles (microscopic modelling) is calculated and then employed to obtain the material response via different homogenization procedures. Ferromagnetic filler particles are usually resolved explicitly or as parts of particle aggregates. Polymer chains can be resolved explicitly (microscopic modeling) or can be represented by an effective medium (mesoscopic modeling). Mesoscopic modeling is employed more frequently as the defining feature of MAE internal structure is the presence of ferromagnetic filler particles, and filler restructuring is the underlying process for the changes in macroscopic characteristics of MAEs. The main aspects of the modeling are the interparticle interactions, equations of motion and collective energy of the system of filler particles and the surrounding polymer. Magnetic interactions are usually described within the framework of dipole approximation, but some works aim to take higher orders of multipole expansion into account. Usually microscopic/mesoscopic models study an element of the material volume to either understand the processes on the scale of a few filler particles or obtain a representative volume element.

#### 3.1.1. Molecular Dynamics Simulations

A special place among combined microscopic/mesoscopic description of MAEs is occupied by molecular dynamics (MD) simulations. This field of study has recently experienced active development.

In spite of obvious simplicity, MD models are able to describe the main features of the magnetic filler restructuring within the polymer matrix under the influence of the magnetic field to explain the microscopic origin of the experimentally observed phenomena.

MD modeling is based on solving the equations of motion of particles that make up the system under study. General patterns and characteristics of the material are derived using the laws of particle motion by calculating the integral properties or considering a representative volume element of the material. To handle the intrinsically multiscale structure of MAEs, namely, the fact that magnetic nanoparticles and especially microparticles are one or even two to four orders of magnitude larger than monomer units of polymer matrix, different level of coarse graining is usually applied. In the simplest approach, only magnetic particles are considered and Langevin equations of either only translational or both translational and rotational motions of the particles are solved:

(1) $$\begin{array}{c} m_{i}\frac{d{\vec{v}}_{i}}{dt}={\vec{F}}_{i}+{\vec{F}}_{i,R}-\xi_{T}{\vec{v}}_{i}\\ I_{i}\frac{d{\vec{\omega }}_{i}}{dt}={\vec{T}}_{i}+{\vec{T}}_{i,R}-\xi_{R}{\vec{\omega }}_{i} \end{array},$$

where ${\vec{F}}_{i}$ and ${\vec{T}}_{i}$ are the total force and torque acting on the particle *i* due to its interaction with other particles, external magnetic field and polymer matrix; $m_{i}$ and $I_{i}$ are the mass of the particle and its inertia tensor. ${\vec{F}}_{i,R}$ and ${\vec{T}}_{i,R}$ are a Gaussian random force and torque, respectively. The last terms account for the translational and rotational friction forces, which are proportional to the particle linear, ${\vec{v}}_{i}$, and angular, ${\vec{\omega }}_{i}$, velocities, with the friction coefficients $\xi_{T}$ and $\xi_{R}$, respectively.

Magnetic particles are usually modeled as beads bearing point magnetic dipoles located in their centers and either freely rotating [30] or firmly connected with the particle body so that the particle rotates as a whole to orient its magnetic moment [31,32,33]. Additionally, it is assumed that the modulus of the magnetic moment is fixed; this approximation works well for either magnetically isotropic monodomain nanoparticles or magnetically hard ones. Due to the presence of a permanent magnetic moment, the particles interact via dipole–dipole interactions:

(2) $$U_{d}\left({\vec{r}}_{ij}\right)=\frac{{\vec{m}}_{i}{\vec{m}}_{j}}{r_{ij}^{3}}-\frac{3({\vec{m}}_{i}\cdot {\vec{r}}_{ij})\left({\vec{m}}_{j}\cdot {\vec{r}}_{ij}\right)}{r_{ij}^{5}},$$

where ${\vec{r}}_{ij}$ is center-to-center vector between *i*-th and *j*-th particles bearing magnetic moments ${\vec{m}}_{i}$ and ${\vec{m}}_{j}$ (the corresponding force is added to ${\vec{F}}_{i}$). Besides, the dipole–field interaction

(3) $$U_{H}\left({\vec{m}}_{i},\vec{H}\right)=-{\vec{m}}_{i}\vec{H},$$

should be taken into account in ${\vec{T}}_{i}$ when an external magnetic field $\vec{H}$ is applied. The later one forces the hard-magnetic particles to rotate in order to orient their magnetic moments along the field lines. In the simulation model proposed in [34], a finite magnetic anisotropy is taken into account via introducing the additional energy of uniaxial magnetic anisotropy depending on the angle between the magnetic moment and the easy axis of the particle. In this case, the rotation of the particle magnetic moment under the influence of the applied magnetic field is affected by both polymer matrix and by internal magnetic anisotropy.

To model a pure repulsion between all beads in the system due to excluded volume, truncated and shifted Lennard–Jones potential (so-called Weeks–Chandler–Andersen potential [35]) is commonly used.

**Figure 3:**
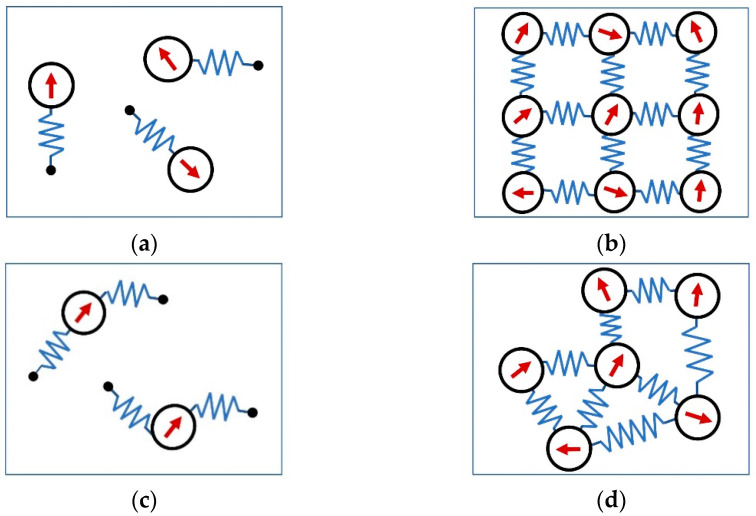
Elastic springs connecting magnetic particles to one (**a**) or two (**b**) anchoring points; elastic springs connecting centers of magnetic particles forming a regular (**c**) or irregular (**d**) network. Red arrows denote magnetic moments of particles.

In the coarse-grained MD models (Figure 3), polymer matrix is usually represented by elastic forces acting on magnetic particles, in addition to magnetic forces and excluded volume interactions. In [36,37], the magnetic particles are connected by elastic springs only to some anchoring points in space, fixing initial positions of the particles. Within this approach, either only translations of the particles can be constrained (one-spring model, Figure 3a) or both translations and rotations of the particles are hindered by the polymer matrix (two-spring model, Figure 3b). In [23], the mechanical constraints acting on magnetic particles due to the presence of a polymer matrix are represented by elastic springs connecting the centers of nearest-neighbor particles. The rigidity of the matrix in these cases can be controlled by the value of the elastic constant in the harmonic spring potential. In more detailed approaches, polymer chains are explicitly modeled as beads on springs, forming either a regular network (Figure 3c) with magnetic particles occupying all [31,32,33] or a certain fraction of crosslinks [34,38], or a non-regular network (Figure 3d) with some beads acquiring magnetic moments and thus mimicking magnetic particles [39,40,41]. The fraction of magnetic beads can be varied but the total amount of particles in this case increases considerably (due to additional beads representing segments of the chains), making the simulations much more time consuming, however, in a sense more realistic, in particular, in the description of magneto-responsive behavior of magnetic gels capable of a large variation of volume.

##### MD Simulations of Magnetic Gels

A lot of efforts have been directed to apply MD simulation technique within the frameworks of simple approaches described above to the study of the structural and conformational behavior of the so-called magnetic macro-, micro- or nanogels, i.e., polymer networks swollen with a solvent and containing some fraction of magnetic nanoparticles. Magnetic gels, or ferrogels, are very promising for biomedical applications, in particular, as drug delivery systems [42]. First models of magnetic gels were quite simple. They were constructed by placing the magnetic nanoparticles on a regular spatial lattice (squire in 2D or simple cubic or diamond cubic in 3D) and connecting them by bead-spring polymer chains attached to specific spots on the surface of the magnetic particles. Periodic boundary conditions were used while the box size was settled in the course of the system equilibration. In this simple approach, in addition to the excluded volume, only dipole–field interactions were taken into account while dipole–dipole interactions were neglected owing to low concentrations of the magnetic particles. As a result, the gel deformations in a magnetic field were explained by a direct coupling of the orientational degree of freedom of the magnetic moments of the nanoparticles to the polymer chains, whose ends were firmly connected with particle surface, creating stress in polymer chains due to rotations of the magnetic particles. It was found that in 2D the particle rotation causes isotropic shrinkage of the gel [31,32] while in 3D the deformations are anisotropic—a strong shrinkage was observed in the direction parallel to the field while the shrinkage in the perpendicular directions was either small or not present at all, depending on the network topology [32,33].

MD simulations of single magnetic nanogels (MNG) with a small fraction of magnetically anisotropic nanoparticles occupying some crosslink beads of a regular polymer structure with equal length of the network subchains were carried out in [34,38,43]. In these papers, not only dipole–field but also dipole–dipole interactions were calculated explicitly owing to the finite size of the system. Besides, the magnetic moment was coupled inside the particle with the easy magnetization axis. The calculated radial distribution functions for varying strength of interparticle dipolar interaction, concentration and temperature clearly indicated the structuring of magnetic particles in the magnetic field. The effect of the particle magnetic anisotropy on the magnetic structures and volume changes of MNGs in magnetic fields was elucidated.

In a series of publications [39,40], the model of irregular polymer network with a fraction of magnetically hard nanoparticles with “frozen-in” permanent magnetic moments was used to investigate the equilibrium structural properties of not only a single magnetic nanogel [39], but also MNG suspensions in absence [40] and in the presence [41] of an applied external field. It was found that inside a single MNG, magnetic nanoparticles form small clusters whose shape is largely affected by polymer elasticity, in particular, the amount of crosslinks [39]. In suspension, MNGs can aggregate due to magnetic interactions leading to formation of magnetic nanoparticle bridges between MNGs [40]. Such self-assembling behavior is largely enhanced when an external magnetic field is applied. Furthermore, it was found that suspensions of MNGs have larger susceptibility to magnetic fields than suspensions of magnetic nanoparticles at the same mean concentration due to a high local concentration of the latter in regions inside the gels [41]. On the other hand, a gel itself has a lower susceptibility than the suspension of magnetic particles of the same concentration due to elastic constraints acting on the particles within the gel. In [44], the behavior of a MNG in the shear flow is studied with the use of the same model.

##### Refined MD Models of MAEs

In general, harmonic spring potentials acting on magnetic particles can describe qualitatively well elastic deformations arising upon particle movements under the action of the external magnetic field and elucidate the role of magnetomechanical coupling in the resulting magnetic structures and some features of MAE magnetization. In [36], a simple model of a magnetoactive elastomer filled with magnetically hard particles was proposed to study the role of inelastic microstructural matrix deformations induced by magnetic fields. This work was inspired by experimental observations of a substantial change in the magnetic response of MAEs containing hard magnetic particles after their first exposure to external fields—initial magnetization curve of these materials differs substantially from the subsequent ones in consecutive measurements of conventional magnetization loops. It was proposed to model irreversible relaxations of elastic constraints during the first magnetization of the sample simply by shifting the anchoring points of the elastic springs undergoing large deformations upon particle movements. This shift reduces the extension of the spring constraining the particle and facilitates its movement during the second magnetization-demagnetization loop. It was shown that only the model taking into account both translational and orientation irreversible constraints is able to describe the experimental observations qualitatively well.

A special approach to studying structural transformations in MAEs filled with non-spherical flake-like NdFeB particles was proposed in a recent paper [45]. In the developed MD model, the magnetic particles are represented by 14 spherical beads rigidly connected to a central bead, thus, forming an anisotropic ellipsoid-like (or flake-like) aggregate. The central bead acquires a magnetic moment which is directed perpendicularly to the flake plane. Anisotropy in mechanical response, i.e., in translation and rotation of the anisometric particles along long and short axes, arises due to different values of the elastic constants for the harmonic springs connecting four non-magnetic beads (per two beads at long and short axes) in each flake-like aggregate to some anchoring points located in space. Furthermore, irreversible deformations under the influence of the magnetic field were modelled by shifting anchoring points. Computer simulations were performed for a fixed value of volume fraction (0.08) of magnetic particles corresponding to the experiments but different values of magnetic moments of particles and rigidity constants of harmonic springs. In spite of the model simplicity (the particles are regular and monodisperse), the model is able to capture the main features of MAE response to moderate and strong fields which are observed experimentally [45]. In particular, it was shown that in a sample pre-magnetized in a strong magnetic field for a few minutes, further application of a moderate magnetic field leads mainly to flake rotations that are fully reversible. In contrast, in initially non-magnetized sample translations and rotations of the flake-like particles in a moderate magnetic field cause non-reversible formation of chain-like structures.

**Figure 4:**
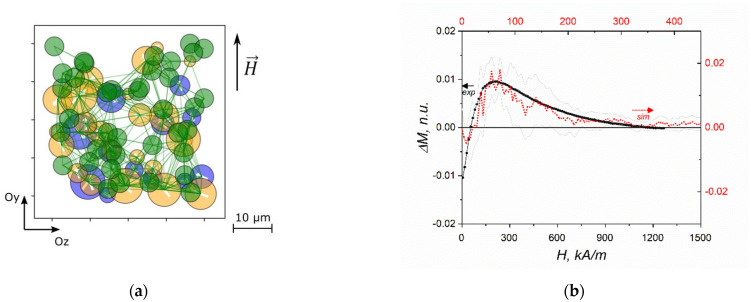
(**a**) Snapshot of the system of ferromagnetic and ferroelectric particles in the simulation box demonstrating an MD model of a multiferroic material: blue spheres are FM particles, orange ones are FE particles, white arrows show their magnetic/electric polarizations, green spheres are polymeric beads, green lines denote elastic links; the snapshot region is 10 × 50 × 50 μm along Ox, Oy, and Oz axes, respectively. (**b**) Magnetization increment $∆M\left(H\right)=M{\left(H\right)}_{E}-M{\left(H\right)}_{0}$, where $M{\left(H\right)}_{0}$ and $M{\left(H\right)}_{E}$ are magnetizations in normalized units measured without electric bias and under an electric bias of 5 MV/m; experimental data (black) and simulation data (red) with error bars (grey) [30]. *H* denotes the magnetic field strength.

In recent papers [30,46], the MD model of a multiferroic material, namely an elastomer matrix filled with both ferromagnetic (FM) and ferroelectric (FE) microparticles was proposed. In comparison with previous approaches, the polydispersity of FM and FE particles was taken into account with lognormal distribution of sizes, and the magnetic and electric moments prescribed to the corresponding particles were scaled according to their size. Polymer matrix was modeled in the simplest way via introducing elastic springs connecting each particle with FM or FE particles inside a sphere of a given radius (Figure 4a). Dipole-dipole interactions were calculated only between particles in the close vicinity. It was shown that when a magnetic or electric field is applied, the corresponding FM or FE particles are moved from their initial positions causing mechanical stresses in polymer matrix to be transferred to the particles of the different type. This kind of particle coupling through polymer matrix was shown to be the fundamental mechanism of multiferroic behavior of the composite, i.e., a magnetization causes an electric response while an electric polarization leads to a magnetic response. The simulation results were confirmed experimentally for a polymer-based dispersion of iron and lead zirconate micrometer-size particles (Figure 4b).

MD approach makes it possible to study not only rearrangement of magnetic particles in bulk but also on the surface of MAE films. In [23], the coarse-grained MD model was applied to study the structure of a 3D thin film of magnetoactive elastomer adsorbed on a solid substrate. Within this model, a MAE film was represented as soft-core spherical magnetic particles, carrying point dipoles, connected by elastic springs. The concentration of magnetic particles as well as the rigidity of the polymer matrix (i.e., values of the elastic constants of the harmonic spring potentials) were varied. The magnetic field was applied perpendicular to the film surface. The equilibrium structures formed by the magnetic particles in magnetic fields were a result of the competition between dipole–dipole, elastic and dipole–field interactions. It was shown that the surface roughness increases strongly with growing magnetic field due to aligning of magnetic aggregates with the field and formation of mountain-like profiles on the film surface. The effects of the concentration of magnetic particles and rigidity of the polymer matrix were elucidated. The obtained results provided some guidelines for fabrication of MAE coatings with a tunable surface topology.

##### Stress-Strain Behavior and Elastic Modulus of MAEs via DPD Simulation

MD models of MAEs mentioned above can capture the structural and magnetization features of magnetoactive polymer materials. In a recent paper [47], the so-called dissipative particle dynamics (DPD) was first applied to study mechanical properties of MAEs. DPD is a coarse-grained molecular dynamics simulation method widely used in modeling of various polymer systems including elastomers [48,49]. This mesoscale method makes it easy to cover much larger time and length scales in comparison with conventional MD and to achieve an equilibrium state even for very large systems. In [47], to capture the size difference between magnetic nanoparticles and monomer units of polymer chains, the nanoparticles were represented by a set of beads bearing fixed co-oriented magnetic moments not connected to the polymer matrix. During mechanical deformations, such particles can transfer the mechanical load only through excluded volume interactions with the polymer. The polymerization of monomers into a network mimicking epoxy resin was performed using reactive DPD, in the absence and in the presence of external magnetic field. The developed approach allowed to estimate densities of the load-bearing chains in the polymer matrix and to correlate them with the Young’s modulus of the material with isotropic and chain-like distribution of magnetic particles obtained from stress-strain curves. The proposed model also allowed us to elucidate the role of the particle/polymer interface by calculating the elastic modulus tuning the interaction parameters between magnetic beads and monomer units of the polymer chains. Although the magnetic nature of the particles came into play only at the stage of preparation of the system with ordered filler, the developed model lays the foundation for simulations of MAEs mechanical properties in magnetic fields.

To summarize, MD calculations are a powerful tool for studying changes in the material microstructure. They have been particularly successful in investigating structural and conformational behavior of the magnetic macro-, micro- or nanogels in magnetic fields, for example for calculating their magnetic properties and volume changes. The merit of very simple MD models is that they not only allow one to describe the main features of filler restructuring and to explain the microscopic origin of the experimentally observed phenomena but also to establish the foundation for the development of useful approximations.

As far as calculations of MAE properties and behavior are concerned, the theoretical investigations were rather limited, probably because they require large computational resources. However, we believe that MD calculations will gain more importance in the future, while the DPD version seems to be the most promising. In particular, MD models can be generalized to more complicated cases, for example anisometric and/or soft-magnetic particles.

#### 3.1.2. Mesoscopic Structure Modeling: Analytical and Numerical Approaches

The simplest models work with the approximation of a uniform lattice network of ferromagnetic filler particles and with the approximation of magnetic dipole interaction [50,51,52,53,54,55]. The use of the magnetic dipole approximation in modeling, however, leads to noticeable errors for the cases of small distances between particles in relation to their size, which corresponds to magnetoactive elastomers with a high filler volume concentration (more than 20% by volume), where the average distance between ferromagnetic particles inside the polymer matrix has the same order of magnitude as the size of the particles themselves. To describe the pairwise interparticle interaction with higher degree of accuracy, a model with a more complex interpolation interaction potential obtained in the multipole approximation was also proposed [56].

In other cases, the polymer network is modeled as a continuous mechanical medium, the elastic properties of which are described by either linear or nonlinear elasticity theories.

##### Calculation of Elastic Moduli

In [50], the dynamic response of a magnetoactive elastomer in the presence of various magnetic fields is described using a coarse-grained model with a cubic lattice that contains filler particles (magnetic dipoles) as the nodes. The particles are connected by linear elastic springs. The approximation of a uniform isotropic distribution of filler particles in the polymer matrix is used in the model. Additionally, the limiting case of weak magnetic fields, which do not lead to the rearrangement of the filler into chain structures, is assumed. The Langevin-type equations of motion of filler particles are linearized with respect to a small parameter of particle displacements from the equilibrium position. In this paper, the relaxation spectrum of a cubic lattice is calculated, expressions for the dynamic elasticity moduli of the material for various mutual orientations of the magnetic field and the direction of shear deformation are obtained. It is shown that the dependences of the dynamic moduli on the magnitude of the magnetic field at low fields can be represented by quadratic functions.

As far back as 1996, the authors of the work [51] laid the theoretical foundation for studying the chain structures of magnetically active particles and their effects on the surrounding elastic medium in the presence of a magnetic field. It was suggested that the shear modulus of the material is a superposition of the modulus in the absence of a magnetic field and the additional modulus induced by the magnetic field. The pairwise interaction of spherical magnetoactive filler particles with magnetic dipoles located in their geometric centers was considered assuming their relative displacement caused by the shear deformation of the sample. An expression for the magnetically induced part of the shear modulus ΔG was derived using the limit of small deformations:

(4) $$∆G\approx \frac{\varphi M^{2}}{2\mu_{1}\mu_{0}h^{3}},$$

where φ is the volume fraction of particles in the composite, M is the particles’ magnetization, $\mu_{1}$ is the relative permeability of the medium, $\mu_{0}$ is the magnetic permeability of vacuum and the parameter $h=r_{0}/d$ is an indication of the gap between particles in a chain. d denotes the particle diameter and $r_{0}$ stands for the distance between the centers of particles in a chain. The maximum possible value of ∆G for a typical MAE, filled with iron particles, can be estimated by taking φ ≈0.29,  saturation magnetization $M_{s}\approx 2.1\;T$, $\mu_{1}=1,\;h=1.$ This evaluation of (4) gives $∆G_{max}\approx 5\times 10^{5}$ Pa, which is about one order of magnitude lower than experimentally observable values. The primary origin of this discrepancy with the experimental values is clear: the solitary chain model ignores possible magnetic interactions between magnetic particles in different chain-like aggregates and changes in mutual positions of particles in an external magnetic field.

This paper also considered the problem of spatially inhomogeneous magnetization of particles: the influence of the field produced by particles on the magnetization of neighboring particles is characterized by the average magnetization. This took into account the ratio of the size of the magnetically saturated part of the particle volume to the entire particle volume which led to a nontrivial dependence of the additional shear modulus on the magnetic field. A conclusion about the quadratic dependence of the part of the shear modulus induced by the magnetic field on the average magnetization of filler particles was made. In [52], a generalization of this model for the case of interacting magnetic filler chain structures was considered using magnetic dipole approximation. The interaction energy and the shear modulus induced by the magnetic field were also calculated for the distributions of filler particles corresponding to the simple cubic and body-centered lattices. The authors of [57] obtained expressions for the elastic modulus and shear modulus of a MAE sample with an isotropic cubic lattice of filler particles using the linear elasticity model, the magnetic dipole approximation, and the magnetization model described by the empirical Fröhlich–Kennely model:

(5) $$\mu_{Fe}\left(H\right)=\frac{\mu_{ini}+\left(\mu_{ini}-1\right)\frac{H}{M_{s}}}{1+\left(\mu_{ini}-1\right)\frac{H}{M_{s}}},$$

where $\mu_{Fe}$ is the relative magnetic permeability of the filler, $\mu_{ini}$ is the initial relative magnetic permeability and *H* is the magnetic field strength.

An alternative approach to explaining the significant MR effect in magnetically and mechanically soft MAEs has been proposed in the works of Kalita et al. [58,59]. Under MR effect we understand the relative change of the shear storage modulus of an MAE in an applied magnetic field. The explanation is based on the so-called single-particle mechanism of magnetostriction, where the total magnetic anisotropy energy of the filling particles in the matrix is the sum of single particle energy terms [60]. An additional magnetoelastic contribution to the mechanical stress created by the induced magnetic anisotropy counteracts the shear and increases the effective shear modulus of the magnetoactive elastomer when the latter is magnetized. Numerical estimates made for the magnitude of magnetorheological effect (almost two orders of magnitude) were in good agreement with experimental data [59].

**Figure 5:**
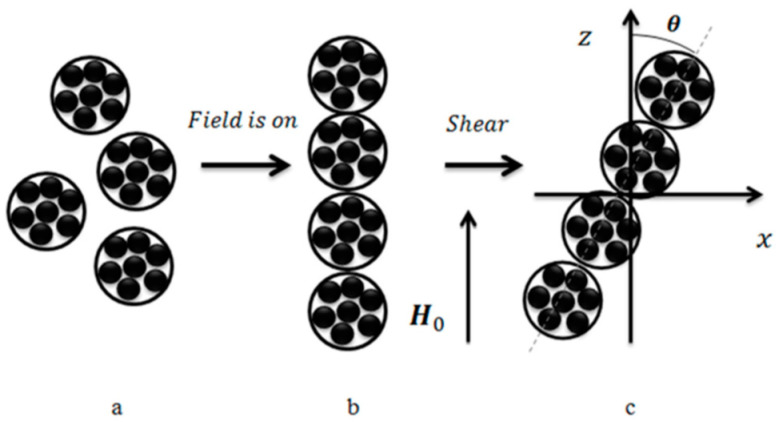
Spherical agglomerates of magnetic particles initially isotropically distributed in polymer matrix (**a**) form chain-like aggregates (**b**) in external magnetic field. Tilting of aggregate chains takes place under simultaneously applied magnetic field and shear deformations (**c**) [61].

**Figure 6:**
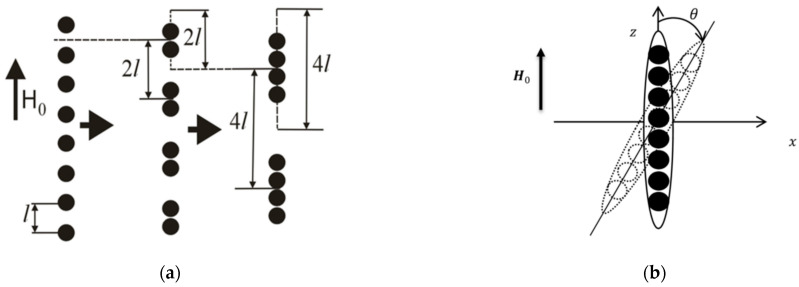
(**a**) Schematic representation of three first stages of the aggregation of agglomerates located on one axis of the lattice parallel to the external magnetic field strength $H_{0}$. The number of agglomerates in the chain aggregate is determined by the balance between magnetic attraction and elastic constraints. Magnetic interactions the agglomerates located on different axes of the lattice are neglected. (**b**) Agglomerate chains are approximated by ellipsoids to describe their tilting under shear deformation [61].

In a series of works, an attempt was made to describe the MR effect in MAEs [61] and magnetic ferrogels [62] quantitatively. A concept of primary aggregates of magnetic particles, first put forward in [63] to explain strong concentration dependence of the shear modulus of alginate ferrogels, was used to catch high values of experimentally measured increase in elastic and loss moduli of these materials in magnetic fields, which could not be described properly considering single magnetic particles dispersed in elastic medium (on the level of single magnetic particles). Isotropic spherical agglomerates of magnetic particles introduced in the proposed model (Figure 5a) had stronger magnetic properties and, in an applied magnetic field, can more easily aggregate into chain-like structures (Figure 5b,c), overcoming elastic forces of polymer matrix than isolated magnetic particles. Furthermore, volume fraction of magnetic agglomerates (including trapped rubber) was claimed to be higher than that of isolated magnetic particles, this fact also favoring magnetic attraction and chaining of the agglomerates in external magnetic field. To calculate the equilibrium aggregation number of chains, a lattice representation was used and a special hierarchical model of aggregation was applied (Figure 6a), taking into account magnetic interactions of agglomerates only within single lines oriented along the field axis. To estimate magnetization of aggregates, they were approximated by ellipsoids of revolution (Figure 6b). In the developed model, it was assumed that primary agglomerates have the same size, and their chain aggregates are monodispersed. Even this crude approximation provided a rather good agreement with experimental results, in particular, it allowed to describe theoretically the high MR response of alginate ferrogels [62] as well as MAEs based on a permalloy filler [61].

The work [64] provided an overview on how to build a bridge from the mesoscopic positioning of the particles relative to each other to the overall, possibly macroscopic behavior of the entire system. To address the MR effect, reduced dipole–spring models were employed. It was found that whether the mechanical moduli increase or decrease under the influence of magnetic interactions depends on the particle configuration and on the orientation of the magnetization direction. Various regular lattices, randomized particle configurations as well as real particle arrangements extracted from experimental samples by X-ray tomography were evaluated. Upon strong magnetization, it was found that a restructuring of the filler takes place. During this process, against the elastic restoring forces of the springs, particles collapse toward each other into virtual contact and form chain-like aggregates. This effect is accompanied by a significant increase in the mechanical stiffness, in qualitative agreement with corresponding experimental observations [65]. The dynamic moduli, quantifying the storage and loss parts of the dynamic response of the systems, were evaluated as a function of the magnetization and for different particle arrangements as well [66,67].

##### Calculation of Magnetostriction

Theory of magnetostriction of MAE samples has received a lot of attention in the literature. The reason is that this phenomenon is important for a number of applications (e.g., actuators for soft robotics), while the comprehensive description of the underlying physics is challenging from the fundamental point of view, even in the case of a spherical MAE sample [68]. If MAE is considered to be a continuous isotropic medium (macroscopic scale), an MAE sphere must stretch along the direction of a uniform magnetic field. On the other hand, taking into account the internal structure of the composite material (mesoscopic scale), one would come to the conclusion that an MAE sphere must contract along the direction of the field, because magnetized particles interact with other particles. As a result, two composites with the same matrix/filler content may behave very differently depending on their mesoscale structure [68].

A qualitative description of the behavior of an elementary spherical cell consisting of a hard magnetic (HM) particle in its center surrounded by an elastic incompressible shell containing a number of uniformly distributed soft magnetic (SM) particles was presented and validated by two complementary theoretical approaches in [69]. These approaches were a continuum analytical description of the magnetoelastic system and coarse-grained MD simulations within a minimal spring-bead model. The main approximations were the linear elastic response and the negligible mutual magnetization between magnetically soft particles. Both models demonstrated that when an external magnetic field is oriented antiparallel to the magnetic moment of the HM particle, a nonmonotonic deformational response of the elementary cell takes place with an increasing field strength. In weak antiparallel fields, local microscopic particle rearrangements cause the shrinking of the cell in the field direction while in stronger fields the elongation along the field axis takes place. MD simulations also provided distributions of SM particles and elastic stresses in the shell depending on the field orientation and its strength.

A theoretical analysis of the effect of magnetic particle concentration on magnetostriction (elongation vs contraction) of an ellipsoidal ferrogel sample in applied magnetic fields was performed in [70]. The change of magnetic free energy under small sample deformations was estimated taking into account both the change of the demagnetizing factor and the magnetic susceptibility. The magnetic susceptibility was calculated assuming linear particle magnetization and pair interaction approximation. It was shown that at particle concentrations below the critical value $\varphi_{crit}\sim 0.162$, contraction of the sample in the field direction can occur. The possibility of this effect has been predicted earlier [71], however, more accurate account of the pair distribution function performed in [70] has shown that the range of the sample aspect ratios, $R_{0}$, where this effect can take place is rather narrow: the samples should be either strongly prolate or oblate. This makes experimental observation of this effect rather rare. In a wide range of $R_{0}$, as well as at particle concentrations $\varphi >\varphi_{crit}$, the sample elongation is more favorable in accordance with experimental data.

In the work [64], the deformation of an MAE sphere in a magnetic field was considered. The particles were assumed to be embedded in a linearly elastic finite-sized sphere. When the particles are magnetized, they distort the surrounding elastic material through the resulting pairwise magnetic attraction or repulsion. Superimposing the contributions of all magnetized inclusions, the overall deformation of the system was calculated [72]. The underlying mathematical expressions were analytical and therefore contained an infinite number of degrees of freedom involved in the distortion of the elastic sphere. The appearance of the global deformation was strongly related to the internal particle arrangement. The shear modulus of the sphere was kept fixed at 1.67 kPa. Therefore, the Young´s modulus of the sphere varied according to the well-known relations between the elastic moduli and the Poisson´s ratio. Whether the sphere was elongated or contracted along the magnetization direction depended significantly on the mutual particle positioning, on the orientation of the magnetization axis, and on the value of the Poisson ratio quantifying the compressibility of the elastic material [72]. For randomized particle configurations, a tendency of sphere´s elongation parallel to the magnetization direction was found, in agreement with corresponding experimental observations [73,74]. More accurate description of magnetostriction phenomenon was performed using a combined micro/meso/continuum approach and described in the corresponding section below.

#### 3.1.3. Mesoscopic Cell Modeling

Due to the fact that the number of filler particles in a real MAE is very large even for the case of low concentrations, the possibilities of direct calculation of material behavior are limited by the computational power of modern computers. One way to solve this problem from a modeling point of view is to consider the properties of a material cell that contains a reasonable number of ferromagnetic inclusions and then calculate or evaluate material properties based on the behavior of this mesoscopic cell. Such smaller systems include single particle cells that help to understand how the presence of ferromagnetic filler influences MAE properties, two particle cells that additionally take into account pairwise particle interactions in the simplest form and multi-particle cells that allow for the introduction of filler distribution-related factors into a model. A notable way of transitioning from a mesoscopic material cell to a macroscopic sample is constructing a representative element of the volume or surface of the material, that is, some element small enough that its behavior can be calculated in a reasonable amount of simulation time, but large enough that the properties and behavior of this element could be related to the properties of the entire macroscopic sample within the specified margin of error. Thus, in the case of studying the MAEs within the framework of the representative volume element approach, it is necessary to construct an element of the polymer medium containing a number of ferromagnetic inclusions corresponding to the filler concentration. At the same time, such an element can be declared as a certain effective “period” of the general internal structure of a magnetopolymer composite. A large number of theoretical studies of MAEs are dedicated to understanding the processes occurring in mesoscopic material cells when external magnetic field and/or mechanical load are present.

The authors of [56] studied the problem of using the magnetic dipole approximation to describe the magnetic interaction of filler particles in magnetoactive elastomers. In order to create a more realistic theoretical model of processes occurring in MAEs, the interaction of a pair of linearly magnetizable spherical particles was studied. In the work, the effective interaction potential for small interparticle distances, as well as the resulting force of magnetic interaction, are obtained. The suggested interaction potential is an approximation of the multipole expansion for the interaction of particles. The equilibrium positions of the two-particle system were found by minimizing the energy functional with the elasticity energy defined by the Mooney–Rivlin model. The hysteresis-type behavior was demonstrated for the equilibrium interparticle distance with a cyclic change in the external magnetic field. A similar modeling process was also used in [75], where the polymer medium was described as a classical medium with properties corresponding to the Kelvin–Voigt rheological model.

**Figure 7:**
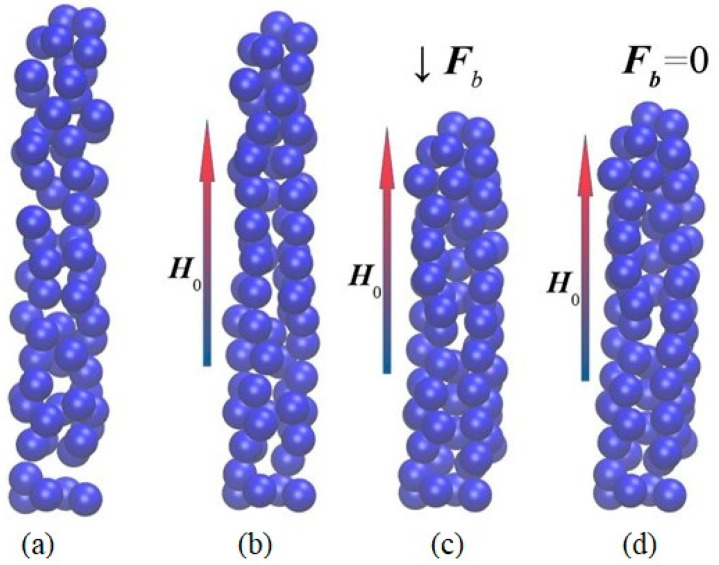
Distribution of the particles in the sample under: (**a**)—the external magnetic field strength $H_{0}=0$ and the end-wall force $F_{b}=0$; (**b**)—$\;H_{0}=10\sqrt{G_{m}}$ and $F_{b}=0$; (**c**)—$\;H_{0}=10\sqrt{G_{m}}$, $\;F_{b}=-6\sqrt{G_{m}}$; (**d**)—$\;H_{0}=10\sqrt{G_{m}}$ and $F_{b}=0$. $G_{m}$ denotes the shear modulus of the matrix [76].

Yu.L. Raikher et al. [76] developed an approach to describing processes on a mesoscopic scale, which makes it possible to calculate the magnetomechanical behavior of the volume element of a magnetically active elastomer in the approximation of the linear theory of elasticity of the polymer medium and the magnetic dipole interaction of filler particles. In this approach, it is assumed that a magnetic dipole is placed in the geometric centers of spherical magnetically soft particles, and the field inside the particle is determined taking into account the demagnetization effect. The magnetic dipole moment of each of the particles in the model depends on the collective magnetic field created by the remaining particles in the selected volume element of the material. Using the particle displacement vectors obtained as a result of solving the finite element problem, the energy of an element of a MAE was calculated, and the equilibrium state of the system was determined via energy minimization. It was shown that the simulated system exhibits pseudoplasticity under the condition of a constant external magnetic field presence and a cyclic mechanical load. Figure 7 demonstrates the calculated pseudoplasticity effect in the loading cycle (a)→(b)→(c)→(d). Initial configuration (a) corresponds to the unloaded sample. The assembly of magnetized particles, when forced to rearrange under pressure, finds a more favorable configuration: under zero mechanical load the total energy of configuration (d) of Figure 7 is lower than that of configuration (b) [76].

In [77] a boundary value problem (BVP) for the composite with mixed filling was considered on a mesoscopic scale: a period of hard magnetic particle chain surrounded by a polymer matrix and soft magnetic particles was modeled using finite element method. In this case, the Langevin function was used to describe the magnetic properties of magnetically hard particles and the Fröhlich–Kennelly function is used to describe magnetically soft medium. The relationship between the mesoscopic model and the macroscopic magnetic characteristics of a MAE with the shape of an ellipsoid was also considered.

The work [78] can serve as an example of the microcontinuum approach with the weak form of the Maxwell and mechanical equilibrium equations determining the behavior of a mesoscopic cell. The authors of [78] calculated the size of a mesoscopic cell with isotropic filler particle distribution that is sufficient for the cell to be a representative volume element. The problem is solved both analytically and using FEM modeling. A further discussion of this work is provided in Section 4 in the context of homogenization procedure.

Considerable effort has been directed towards understanding the physical foundations of magnetization features of MAEs based on HM particles and a mixture of HM and SM fillers (so-called hybrid MAEs). HM particles are usually composed of multiple magnetic domains, and magnetization of mechanically soft MAEs containing HM particles includes two processes: the intrinsic motion of the atomic magnetic moments of the particles caused by their interaction with an applied magnetic field and mechanical rotation of the magnetic moments together with the particle body.

A model that takes into account a complex structure of micrometer-sized HM particles and couples the processes of particle intrinsic magnetization and rotation within the soft viscoelastic medium was proposed in [79]. A spherical HM particle was supposed to consist of a densely packed solid assembly of identical single-domain nanograins with an isotropic distribution of the nanograin easy axes. Magnetization of nanograins was described using the Stoner–Wohlfarth model according to which the energy of a single nanograin can be written in the following way:

(6) $$E_{grain}=-mH\left(\vec{e}\cdot \vec{h}\right)-KV{\left(\vec{e}\cdot \vec{n}\right)}^{2}+E_{mech},$$

where $\vec{e}$, $\vec{h}$ and $\vec{n}$ are unit vectors of the magnetic moment $\vec{m}$, magnetic field strength $\vec{H}$ and the easy axis of the nanograin magnetization, respectively; K is the energy density constant for magnetic anisotropy, V is the grain volume and $E_{mech}$ is the mechanical energy attributed to each grain (which is equal to zero in the discussed model). The total potential energy of a multigrain particle in an elastic medium included the elastic contribution due to the particle rotation, which was accounted for within the linear Hookean approximation and the magnetic contributions, namely the magnetic anisotropy energy, Zeeman interaction with the magnetic field and pair-wise dipole–dipole interactions between all the nanograins. It was shown that due to magnetomechanical coupling, the magnetic hysteresis loop of a particle composed of highly coercive grains progressively shrinks with the increase of the matrix elastic modulus. The developed model was applied to describe the magnetization curves of MAEs based on HM NdFeB particles [80]. The results of the theory are consistent with experimental observations, the proposed theory is able to describe training effect, negative bias, and reduction of coercivity.

Using the same model for HM multidomain particles, the authors of [81] proposed a generalized model of hybrid magnetic elastomers filled with a mixture of HM and SM microparticles. The magnetization of the SM particles was described by the Fröhlich–Kennelly equation while the interaction between the two types of particles was accounted for within the mean-field approach. First-order reversal curve (FORC) diagrams were calculated for different values of the elastic modulus of the polymer matrix. It was demonstrated that the FORC diagrams display specific new features due to interactions between HM and SM phases and matrix elasticity.

To summarize, a significant progress in understanding the underlying physical phenomena in MAEs has been achieved using numerical and analytical approaches to micro/mesoscopic structure modelling. If the early models accounted only for the magnetization process, recent models took additional effects into account, allowing one to explain significant changes in the elastic moduli of soft MAEs (with soft magnetic, hard magnetic and mixed filling), which are closer to experimental values. As far as the deformation of MAE bodies in an external magnetic field is concerned, a general understanding of factors affecting the deformation of simplest bodies (e.g., ellipsoids of rotation) has been reached. In general, the approaches discussed in Section 3.1.1 and Section 3.1.3 allowed us to establish the origin of the observed magneto-mechanical phenomena on the level of the restructuring of particles. From the results obtained by many scientific groups and challenges they faced, it follows that additional work is required to leave the dipole–dipole approximation in modeling of magnetic interactions for highly filled MAEs. Complex microstructures (non-uniform, anisotropic) and filler particle clustering also require more rigorous and comprehensive research. Although the effect of geometric and magnetic anisotropies of filler particles on MAE response to external stimuli has received a considerable amount of attention in recent years, the variety of possible particle shapes and crystal structures of ferromagnetic particles makes it very difficult to reach reasonably complete scientific understanding in this area of studies. Thus, it is expected that future research will be focused on cluster-like filler structures, multidisperse anisotropic fillers and more complex forms of interparticle interactions (both magnetic-field and matrix-mediated).

### 3.2. Continuum Modeling

The most mathematically rigorous approach with well-developed fundamentals is the continuum approach. In the framework of this approach the composite is described as a whole using field equations. Instead of the internal structure of the material, the emphasis is put on its macroscopic response and properties. The underlying theoretical foundation consists of theory of elasticity, physics of magnetic materials and thermodynamics. The main result obtained through continuum modeling is a relation between macroscopic stress and strain tensors taking into account material magnetization. Free energy of the system used to obtain constitutive relations is described as a function of the Cauchy–Green tensor invariants as well as various convolutions of the Cauchy–Green tensor with the magnetic field vector. To construct a continuum model of MAEs it is necessary to obtain the expressions for the magnetic field inside the ferromagnetic phase and the free energy of the material. Analytical solutions of the corresponding magnetomechanical BVPs usually cannot be obtained, therefore the finite element method (FEM) is frequently employed instead. More simple limiting cases are studied rigorously: the cases of small deformations as well as weak magnetic fields.

There are two main ways of creating a continuum model: direct modeling and homogenization-based modeling. The first path requires deriving a full system of field equations that describe mechanical, magnetic and thermodynamic characteristics of the entire sample based on its material properties and behavior. These are so-called “top-down” models. The second path involves averaging the local characteristics of the medium and takes into account the internal structure of the composite. Obtaining explicit analytical solutions for both approaches is very difficult, especially if the general case of arbitrary deformations and magnetic fields is considered. Custom FEM models can provide numerical solutions of the continuum equations; however, more rigorous and universal theoretical frameworks require mathematical description of material behavior. The most prominent approach to material behavior description found in scientific literature involves explicitly characterizing the thermodynamic potentials of the MAE sample, specifically Helmholtz free energy.

#### 3.2.1. Mechanical Engineering Approach

The most natural way of describing the sample’s behavior on a macroscopic scale is the direct solution of equations that describe the displacement of each point of the sample under external load and the influence of the magnetic field. The sample can be described as a solid body (or a system of smaller material volumes) governed by classic mechanics. Most often a direct approach is based on solving Newtonian equations of motion for linear theory of elasticity and Maxwell’s equations. Alternatively, the information about the magnetic part of the problem is contained in the expressions representing forces acting on each volume element or each point. This approach does not capture the fundamentals of magnetomechanical coupling or the mechanisms of filler restructuring in MAEs, however it is useful for practical applications and especially soft robotics, which has seen rapid development over the course of recent years.

One of the most common tools for analyzing the motion of MAE samples is Newtonian mechanics, namely the equations of translational and rotational motion within the framework of linear elasticity. The displacement of individual small elements of the sample can be described by taking into account the influence of gravitational forces, viscous or dry friction forces depending on the surrounding medium, lifting forces in the liquid, magnetic forces, as well as forces created by the shift of adjacent small elements. Calculation of each of the listed forces usually requires additional modeling considerations, experimental data, or numerical analysis.

Another common tool for describing deformation in MAE samples of simple shapes is the Euler–Bernoulli quasi-static theory of beam bending (or the more general Timoshenko–Ehrenfest beam theory [82,83]). Within the framework of this theory, an elongated object is assumed to be one-dimensional, and a fourth-order differential equation that relates the external load and bending at each point of the object under study is derived:

(7) $$\frac{d^{2}}{dx^{2}}\left(EJ\frac{d^{2}w}{dx^{2}}\right)=q\left(x\right),$$

where E is the modulus of elasticity of the sample, J is the moment of inertia, w is the bend at a given point, q is the external force acting per unit length of the sample. In this case, this force is of a magnetic nature, so its distribution along the length depends on the distribution of magnetization in the robot. Depending on the chosen approximations, the basic equation of the Euler–Bernoulli theory is reduced to a differential equation of the third or fourth order. The Euler–Bernoulli equation (or the definition of the bending moment from which it follows) is also used as one of the terms in Newton’s equation of motion to obtain a more complete picture of the displacements of the robot elements. The Euler–Bernoulli theory is quite simple and understandable, and therefore is often used in modeling that does not require a fundamental theoretical study of the processes under consideration. In [84], the Euler–Bernoulli theory was used to explain the bending of MAE cantilever beams with hard-magnetic particles, initially magnetized perpendicularly to the beam’s plane. The magnetic field was applied in the beam’s plane, and it was perpendicular to the initial direction of particles’ magnetization (before bending). Modeling the effects of the magnetic field on the cantilever as a generalized distributed moment worked well as a phenomenological approach [84]. In particular, using an expression for the linear magnetic energy density [85], an ordinary differential equation for the beam deflection was obtained in the small deflection angle approximation. This equation could be solved analytically. An explicit expression for the field-induced beam stiffness showed that it was proportional to the square of the applied magnetic field strength.

A more precise, general and more complex theoretical tool is the Cosserat rod theory [86]. This theory makes it possible to take into account tension, shear, torsion and bending of an oblong body. Cosserat’s theory combines the evolution of the rod geometry (nonlinear process) and the evolution of the mechanical characteristics of the rod (linear process). In the Cosserat model, the rod is a quasi-one-dimensional system described by the curve $\vec{r}\left(s,t\right)$ passing through the centers of the longitudinal sections, parametrized in space using the parameter of the geodesic of the rod s, and evolving in time. The section of the rod at each point is described by an orientation quaternion consisting of local axes of the Lagrangian coordinate system, indicating the direction of the axis of rotation of the section, and the angle of rotation around this axis. The position of the center of the section $\vec{r}\left(s,t\right)$ evolves under the influence of the forces arising in the rod, and the orientation of the section evolves under the influence of the torques arising in the rod. Obviously, the orientational quaternion of the cross section is also related to the rotation of the magnetic moments of the filler particles in the rod. To calculate the necessary forces and torques, the momentum balance equations are used at each point of the rod with the magnetic field serving as an external stimulus. It should be noted that the elastic properties of the rod in the Cosserat theory are described by the linear theory of elasticity. Thus, when using the Cosserat theory, it is necessary to solve a closed system of 13 equations that determine the behavior of small elements of the rod. The analytical solution of such a system is often difficult or even impossible due to the nonlinearity of the geometric relationship between the local Lagrangian and Euler coordinates, so numerical methods are used to obtain results within the framework of the Cosserat theory.

Kalita et al. [87] used the expression for the elastic energy of the deformed thin elastic beam to explain the so-called critical bending of a soft-magnetic MAE induced by magnetic field. This phenomenon is characterized by a critical exponent for the bending magnitude, and the derivative of the function characterizing the bending has a singularity in the critical region.

An important basic functional element of many actuating devices is an active soft membrane. Such membranes are used as pumps, filters and as elements of devices that allow for remote-controlled handling of liquids. Membranes are a specific case of thin systems, and, as such, it is possible to develop theoretical descriptions of membrane-based devices that include analytical solutions of the boundary value problems of MAE behavior in external magnetic fields. The work [88] made use of both coarse-grained MD simulations as well as continuum modeling to study the influence of precessing magnetic field on the magnetodeformation of a membrane consisting of a single layer of superparamagnetic colloid particles for varying precession angle of the magnetic field. It was shown that the ratio of the magnetic constant to the elastic constant defines the deformation mode in the system under study. The work [89] developed the membrane theory for MAE-based devices. The asymptotic expansion of variational equations of 3D continuum theory was used to obtain an effectively two-dimensional theory of membrane deformation. Both stress and deformation profiles of circular and annular membranes were obtained for different magnetoelastic loading conditions. The model was also validated using existing data from literature.

Finally, another generally accepted approach to describing MAE behavior is finite element modeling using linear continuum field equations of mechanics and magnetostatics [14,90,91,92,93,94,95]. This is the most direct macroscopic approach to the description of physical processes. The stresses arising in the robot are divided into mechanical (of an exclusively mechanical nature) and magnetomechanical (induced by a magnetic field). The latter are calculated by solving Maxwell’s equations. Then the balance equation of the total stress is solved while taking into account the influence of external forces. Since finite element calculations for complex systems in three-dimensional space require significant time and computational resources, they are usually limited to the study of two-dimensional models that qualitatively describe the real movement of the sample. The use of linear theory is also caused by the duration of calculations for non-linear models. The advantage of this approach is the clarity and the ability to set an arbitrary configuration of the magnetic field, as well as the geometric characteristics of the sample and determine which system has the properties necessary for the expected practical applications. Another advantage of finite element modeling is the existence of ready-made software packages that implement the computational foundations of the method. It is then possible to build and optimize a specific model without the need to create new software from scratch.

Thin MAE rods can be said to be a type of system suitable for direct modeling as well as various simplified models. Dimensional reduction procedure can be carried out for such systems. This reduces the complexity of the problem by modeling the MAE as a one-dimensional system. The main modeling assumption in this case is that any material vector that was normal to the rod centerline in the undeformed configuration remains normal to it and does not experience stretching after deformation occurs. This naturally limits the model to describing simple bending but allows for much easier analytical study of MAEs. The works [96,97] studied MAE rods with saturated magnetically hard filler in the presence of both uniform and gradient magnetic fields. The virtual work principle and Kirchhoff-like equations of motion for rods were used and modified to include magnetic torques and forces. Long-range magnetic interactions were neglected and the absolute value of magnetization of different parts of the rod did not change. MAE deformation and displacement was modeled. In [96] results obtained for simple beams were extensively compared with both experimental data and full-field 3D FEM modeling. In [97] regular rods and helical MAEs were considered analytically, numerically and experimentally. The deformational behavior of the material in magnetic fields obtained theoretically was shown to be in good agreement with experimental data. Models obtained through dimensional reduction were shown to describe simpler MAE systems adequately and can thus be used to study prolate MAE samples with high degree of symmetry more efficiently.

In [98] field-induced vibrations of a rod-shaped MAE sample fixed at one end were studied. A numerical solution of vibration equations was obtained using commercial FEM software ANSYS^®^ Workbench 16.2, analysis system “Modal”.

In [90], thin elastomer samples containing magnetically hard particles were studied. Different areas of the samples had different preferred directions of magnetization. Finite element modeling was performed in the ANSYS^®^ software package with MATLAB^®^ scripts by dividing the sample into sections, each of which is considered to be a magnetic dipole with the deformation of each section described using the beam theory of Euler–Bernoulli. In [91], worm-like MAE samples were considered by dividing them into segments, each of which has its own direction of magnetization. Here samples with both hard magnetic and soft magnetic filling were studied. The proposed theoretical model resembles a simple polymer chain model in which the elastic and magnetic moments at the ends of the segments are balanced using an iterative process. The obtained material behavior largely coincides with their experimental behavior, although the system was not described in detail.

In [99], a cuboid sample of the MAE was studied. Silicone elastomer was used as a polymer matrix, and NdFeB particles were used as filler. The MAE under study had an inhomogeneous magnetization profile: the distribution of the magnetic moment direction along the length of the sample was described by a harmonic function. The authors of this paper proposed to use oscillating magnetic field with spatially homogeneous components $B_{x}$, $B_{y}$, $B_{z}$ to rotate the sample and change its shape. This was used to create movement of the MAE sample in a surrounding liquid medium or allow it to bypass various obstacles, thus effectively creating a remotely controlled soft robot. Gradient magnetic fields were also considered. The bending of the MAE is described by solving the equations of the Euler–Bernoulli theory for a rod with free ends, where the distributed magnetic moment acted as the stimulus that induces bending of each section of the rod. Using the energy conservation, the kinematic parameters of the sample’s movement caused by successive controlled changes in its shape were calculated: the maximum height of the “jump” from a flat surface, the speed of rolling along the surface, the speed of horizontal movement (“walking”), the magnetic field required for climbing onto the water meniscus, swimming speed in liquid medium. When studying the floating of a sample in a liquid, the analysis of the mechanical natural frequencies of the sample was carried out by solving the equations of Newtonian mechanics for the rod element using separation of variables. As a result, within the framework of classical mechanics, as well as the quasi-static theory of Euler–Bernoulli beam bending, equations describing the motion of a simple MAE-based soft robot in a liquid medium and in the air were obtained and solved (analytically for linear and numerically for nonlinear cases). Dependences of the kinematic parameters of various types of motion on the dimensions of the sample, as well as on the amplitude and frequency of the external magnetic field were provided. Experimental video measurements of the characteristics of the shape and movement of the robot for various geometric parameters of the sample were carried out. Comparison of simulation results with experimental data showed the adequacy of the proposed models for all types of motion except for swimming.

In [92], FEM was used for active origami-inspired designs, which incorporated active materials such as electroactive polymers and MAEs into self-folding structures. Constitutive relations were developed for both electrostrictive and MAE materials to model the coupled behaviors explicitly. Shell elements were adopted for their capacity of modeling thin films, relatively low computational cost, and ability to model the intrinsic coupled behaviors in the active materials under consideration. The electrostrictive coefficients were measured and then used as input in the constitutive modeling of the coupled behavior. The magnetization of the MAE was measured and then used to calculate the magnetic torque as a function of the special orientation, which led to spatial deformation of the MAEs. Through quantitative comparisons, simulation results showed good agreement with experimental data.

The authors of [93] studied the behavior of a jellyfish-like device consisting of a magnetoactive polymer core and “tentacles”, the ends of which are non-magnetic. The device was placed in a liquid medium in the presence of an oscillating magnetic field. Based on the analysis of the video of the movement of the device, the kinematic characteristics of the jellyfish were calculated, and a simple dynamic simulation of the movement of the tentacles as a rotation of a sequence of small elliptical cylinders around the attachment point of the tentacle was also carried out, the speed of the device is calculated by integrating the Newtonian equation of motion. The results of the calculation of the average velocity were consistent with the experiment for brief time periods of motion. The work also evaluated the influence of the geometric dimensions of the device’s components on its behavior using the Euler–Bernoulli theory and two-dimensional finite element modeling via the COMSOL Multiphysics^®^ 5.3a software package.

In [94,95], systems of cilia-like samples were studied: soft cylinders made of a magnetically active material, fixed at one end on a specific surface. The collective motion of an array of cilia in a liquid medium and in the presence of a magnetic field was studied, taking into account their hydrodynamic interaction. In [94], magnetite was used as a magnetoactive filler, and cilia had sizes of the order of tens of micrometers; in [95], NdFeB particles acted as filler, and cilia had sizes of the order of millimeters. Such systems are capable of generating flow and waves in a fluid both for the purpose of moving external objects and for the purpose of moving the device to which they are attached. In [94], the behavior of the system was described by calculating the configuration of the magnetic field and fluid flow via the finite element method and using the obtained data to calculate the deformation of cilia according to the Euler–Bernoulli theory. In [95], the finite difference method and the Cosserat rod theory were used instead.

To summarize, the approach based on the technical mechanics is pragmatic and application-oriented: it is not focused on revealing the underlying physical phenomena within the composite material but addresses the actuation of MAE-based functional elements. The properties of constitutive materials have to be known. We believe that the combination of microscopic and mesoscopic modeling, as described in preceding sections, with the methods of technical mechanics will lead to rapid development of a fit-for-purpose MAE material design.

#### 3.2.2. Invariant Theory

**Figure 8:**
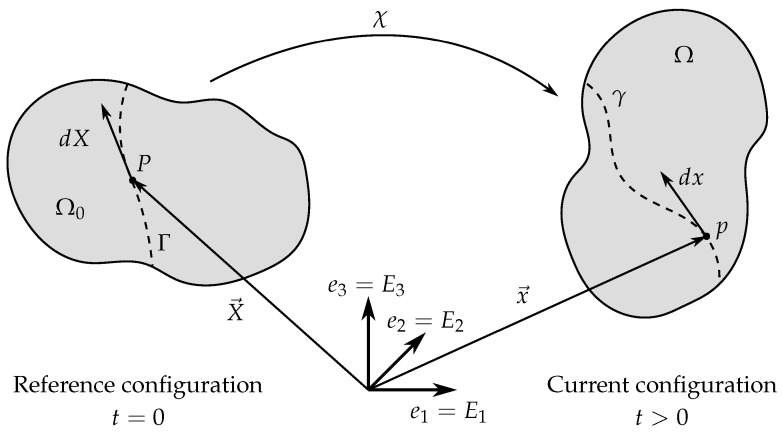
Visualization of different descriptive approaches for the movement and deformation of a continuum body in space by two selected configurations in time (t=0 is the reference configuration, t>0 is the current configuration). Describing a movement or deformation relative to the coordinates of a reference configuration (undeformed) is called the Lagrangian description, while describing it relative to the coordinates of a current configuration (deformed) is called the Eulerian description [105].

The fundamentals of the continuum approach to the theoretical description of magnetoactive elastomers were comprehensively described in [100,101,102,103]. All basic equations were provided in their general form, additional conditions and material relations were also given. These papers described the mathematical structure of the desired functions corresponding to the energy, mechanical, and magnetic characteristics of the material in terms of Lagrangian and Euler coordinates, magnetic field, and Cauchy stress tensor invariants (Figure 8). The results were obtained both directly from the balance equations for the mass, momentum and energy in the Euler configuration, and from the minimization of the energy functional in the Lagrangian configuration. The invariant theory for tensor fields in continuum mechanics was described in great detail in the book [104].

(8) $$\begin{array}{ccc} F=\frac{\partial \vec{x}}{\partial \vec{X}} & J=detF & C=F^{T}F \end{array},$$

Here F is the deformation gradient tensor, $\vec{x}$ are the coordinates in current (Euler) configuration, $\vec{X}$ are the coordinates in reference (Lagrangian) configuration, C is the right Cauchy–Green stress tensor.

Maxwell equations for a stationary case with no free currents (classic magnetostatics):

(9) $$\begin{array}{cc} div\vec{B}=0 & curl\vec{H}=0 \end{array},$$

Here $\vec{B}$ is the magnetic flux density (or *B*-field) and $\vec{H}$ is the magnetic field strength (or *H*-field).

The magnetomechanical balance equation reads:

(10) $$divP+\vec{f}=0,$$

where P is the reference total stress (both mechanical and magnetic), represented by the first Piola–Kirchhoff stress tensor, and $\vec{f}$ is the total force acting on the material volume.

Magnetoelastic free energy is considered as a function of the magnetoelastic invariants: there are six invariants in the case of an isotropic internal structure of the composite and there are ten invariants $\left(I_{1},I_{2},\ldots ,I_{10}\right)$ when structural anisotropy (transverse isotropy) is taken into account [102].

Additionally, the free energy function is composed of several terms: corresponding to isotropic and anisotropic material mechanical contributions, purely magnetic contributions and coupled magnetomechanical contributions:

(11) $$\begin{array}{c} \Psi =\Psi \left(I_{1},\ldots ,I_{10}\right)=\Psi_{iso}\left(I_{1},I_{2},I_{3}\right)+\Psi_{aniso}\left(I_{7},I_{8}\right)+\Psi_{mag}\left(I_{4}\right)+\\ \Psi_{couple}\left(I_{5},I_{6},I_{9},I_{10}\right), \end{array}$$

Coupling terms depend on the coupled invariants and are the most sophisticated aspect of this approach. Another way of constructing the free energy expression is dividing it into the polymer matrix energy, the filler particles energy and the energy corresponding to the interaction between them. The simplest case of small deformations and weak magnetic fields would lead to a free energy function with a quadratic dependence on the magnetic field and quadratic dependence on Cauchy strain. In general, the correspondence principle should be satisfied for the free energy expression as it should correspond to classic elasticity and magnetostatics in the limits of infinitesimal strains and weak magnetic fields, respectively, as those limits imply that the coupling effects practically vanish. The purely mechanical part of the energy stored in the medium most often takes either linear elastic form or hyperelastic form (neo-Hookean, Mooney–Rivlin, Gent, etc.), while the magnetization energy function is usually based on linear, Langevin, hyperbolic tangent or Fröhlich–Kennelly models. The arguments of these model functions are themselves functions of magnetomechanical invariants. The limiting cases of small deformations as well as weak magnetic fields and saturation-level magnetic fields provide the asymptotically correct forms of energy expressions, and the derivatives of mechanical and magnetic parts of the energy correspond to the linear elastic modulus and magnetic permeability, respectively.

Mathematical expressions for the invariants using one of the notations are as follows:

$$\begin{array}{ccc} I_{1}=trC & I_{2}=\frac{1}{2}\left({\left(trC\right)}^{2}-trC^{2}\right) & I_{3}=detC \end{array}$$

$$\begin{array}{ccc} I_{4}=\vec{B}\cdot \vec{B} & I_{5}=\vec{B}\cdot C\cdot \vec{B} & I_{6}=\vec{B}\cdot C^{2}\cdot \vec{B} \end{array}$$

$$\begin{array}{cc} I_{7}=\vec{N}\cdot C\cdot \vec{N} & I_{8}=\vec{N}\cdot C^{2}\cdot \vec{N} \end{array}$$

(12) $$\begin{array}{cc} I_{9}={\left(\vec{B}\cdot \vec{N}\right)}^{2} & I_{10}=\left(\vec{B}\cdot \vec{N}\right)\left(\vec{B}\cdot C\cdot \vec{B}\right) \end{array},$$

where ere $\vec{N}$ is the unit vector describing a specific preferred direction that exists in the material due to its internal structure. This set of invariants describes a transversely isotropic material. If there exists another preferred direction with its own unit vector, then additional similar invariants are introduced.

The constitutive equations for the material (as described by the Coleman–Noll procedure [106]) are as follows:

(13) $$\begin{array}{cc} P=\frac{\partial \Psi }{\partial F} & \vec{H}=\frac{\partial \Psi }{\partial \vec{B}} \end{array},$$

Finally, one needs to add the material equation for magnetic materials:

(14) $$\vec{B}=\mu_{0}\left(\vec{H}+\vec{M}\right),$$

where ere $\mu_{0}$ is the magnetic permeability of vacuum and $\vec{M}$ is the magnetization vector. Boundary conditions are required to complete the problem formulation.

It should be noted that all Maxwell and balance equations must hold for both reference and current configurations. There are several different formulations of the problem that rely on different basic variables: $\vec{H}$ and $\vec{B}$ can be interchanged via partial Legendre transformation; right and left Cauchy–Green tensors are used for reference and current configurations, respectively. Magnetization can also be used as a magnetic variable.

A simple continuum model based on invariant theory was presented in [107]. It utilized finite deformation theory and free energy of a neo-Hookean solid with saturated magnetically hard filler and was tested in ABAQUS 2016 via uniaxial prism deformation and beam bending. The remanent B-field used in the free energy was measured experimentally for all the samples. The results for small bending were obtained both analytically and numerically, and the agreement presented in this work was shown to be good. Finite-element modeling for more complex 2D and 3D structures was compared with experimental results, and it can be said that the general material behavior was captured by the model correctly. The main advantages of this model are its relative simplicity and the ability to predict bending-type behavior of hard magnetic MAE-based regular structures to a reasonable extent.

In [102], an analytical form of the stress-strain relation was derived under the condition of a linear dependence of the MAE free energy on the stress tensor invariants. In [100,103], the case of a polynomial dependence of the free energy on the invariants of the Cauchy tensor was considered. In [108], a polymer matrix containing cylindrical ferromagnetic inclusions was studied, and the strain energy density function was described by the Gent hyperelastic model. Additionally, various specific forms of the free energy expression were proposed and obtained within the phenomenological framework [102,109,110]. In [105], a much more complex form of free energy was used in combination with finite element modeling.

MAE magnetostriction was modeled in [111], where governing equations of the stationary magnetic and the coupled mechanical BVPs were provided. The stationary magnetic BVP was given by Maxwell´s macroscopic equations. Coupled mechanical BVP contained an additional body force density in the coupled magnetomechanical case; this approach comes from the book of de Groot and Suttorp [112] and results from a distinction between long- and short-range contributions of atomic interactions, which is not free from arbitrariness. Constitutive model was based on the works of Dorfmann and Ogden [103], Eringen and Maugin [113], Spieler et al. [114] and Vogel et al. [115]. Since small strains and nonlinear magnetizations were considered, restrictive conditions were introduced on the magnetization and the total stress tensor. The mechanical part of the specific free energy was characterized by an isotropic Hookean materials law. Magnetization was described phenomenologically by the hyperbolic tangent model. Computational homogenization used macro-homogeneity condition. Idealized lattices as well as compact and wavy chains were considered. Random microstructures, comprising both unstructured (random heterogeneous) and structures (chain-like) arrangements, were considered. Qualitative agreement between theory and experiments was demonstrated.

**Figure 9:**
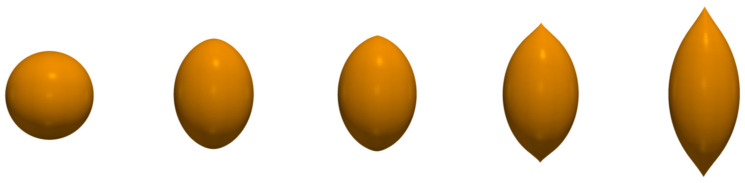
Shape evolution of a MAE sphere with the magnetic susceptibility χ = 0.2 under increasing magnetic field; the nondimensional magnetic field strengths are (from left to right): ${\bar{H}}_{0}$ = 0; 5.7; 6.12; 7.42 and 9.55. ${\bar{H}}_{0}$ is defined as ${\bar{H}}_{0}=H_{0}/\sqrt{G},$ where G is the MAE’s shear modulus [116]. Note that the normalization is performed in the cgs system of units.

In the work [116], the resulting shape of an initially spherical MAE sample was studied. The sample was considered to be linearly magnetizable, isotropic and hyperelastic. Magnetostriction under the influence of external uniform magnetic field was simulated using FEM. The results were compared with the sample elongation predicted by perturbation theory. It was noted that the leading factor that affects the sample behavior is the sample shape and that it was possible to predict complex shape changes without considering the microstructure of the sample. The results obtained through modeling indicated that a spherical sample changes into an ellipsoid and then into a spindle-like object as the external field strength increases (Figure 9). An interesting result presented in this paper is that even simple, initially spherical MAE samples obtain a complex shape due to magneto-deformation in uniform fields, which is an important factor to consider for further research.

The works [117,118] studied the material response to external magnetic field and deformation within the framework of invariant theory in addition to dipolar mean-field theory that combines microstructural and macrocontinuum models and is discussed in Section 4.3 in more detail. The microscopic effects were not discussed in [117,118], and instead authors focused on the role of the initial (undeformed) shape of the sample in the material behavior. MAEs with random isotropic filler particle distribution were considered, and one of the main assumptions of the proposed model was that, under the influence of applied magnetic field, the initially isotropic MAE becomes transversely isotropic through the formation of anisotropic filler particle structures. Thus, the free energy of the sample was divided into isotropic term, anisotropic term and magnetic term with the magnetic part of the energy depending on the sample shape. Uniaxial deformations were studied in [117] while ref. [118] focused on shear deformations. The strong effect of the MAE shape on the sample behavior was demonstrated even within the modeling limitations of an ellipsoidal sample.

The works [119,120] tackled the problem of analytical description of MAEs with two types of magnetic filler: rigid iron particles and soft ferrofluid particles. Explicit free energy function for isotropic MAEs with such fillers was constructed for the two-dimensional and three-dimensional cases in [119] and then used in [120]. The polymer matrix was considered to be incompressible and non-Gaussian. The filler particles were described using classic non-linear magnetization functions (Langevin model and Brillouin model). The variational problem for the free energy was considered using analogies with results obtained before for electroelastic systems by the same authors in [121,122]. Approximate solution of the problem was obtained, and it was demonstrated that it is asymptotically exact for the case of small deformation and weak to moderate magnetic fields. Macroscopic magnetoelastic response (namely, deformation gradient tensor) of suspensions of circular (2D) and spherical (3D) particles was obtained in [119] and compared with full-field FEM simulations. Spherical and cylindrical MAE samples were studied. It was noted that MAEs with ferrofluid filler exhibit stronger magnetostriction effect than their iron-filled counterparts. Discrepancies between theoretical results and experimental data were noted for the case of cylindrical elastomer samples. In [120] the authors proposed an approximate solution that is asymptotically exact for both weak and strong magnetic fields and compared the obtained results with FEM simulations for spherical MAEs with iron and ferrofluid particles. The comparison proved the adequacy of the suggested model based on invariant theory.

Authors of [123] suggested modifying the isotropic part of the mechanical energy and provided a constitutive model with exponential-logarithmic dependence of the energy on the first invariant $I_{1}$. Both isotropic and transversely isotropic materials were modeled, and for the sake of simplicity the dependence of the energy on the purely magnetic response of the material was modeled as additional shear modulus that is exponentially dependent on the applied magnetic field. This is an approach that is somewhat similar to rheological modeling, and it simplifies the calculations. Stress-strain curves and magnetorheological response of MAE samples were obtained, and while the results agree with experimental data for filler concentration around 10 vol% and weak to moderate magnetic fields, a noticeable discrepancy between modeling predictions and experimental data is observed for a filler concentration of ~20 vol% and a magnetic field of about 1 T.

The work [124] presented an alternative continuum modeling framework that was based on the usage of spectral invariants of the magnetomechanical medium instead of classic invariants. These spectral invariants consist of the eigenvalues of the Cauchy–Green tensor (which can be interpreted as principal stretches of the system) and traces of different tensors obtained as the products of vectors that define the preferred directions of the material: external magnetic field direction, initial filler structure anisotropy and Cauchy–Green tensor eigenvectors. There are ten independent spectral invariants for a transversely isotropic material, and they can be expressed in terms of classic invariants. The main advantage of this framework is the ability to vary each spectral invariant independently in a triaxial stretch test and obtain the dependence of the free energy of the system on each invariant directly. This approach presented promising and logical direction for further theoretical studies of MAEs and other magnetosensitive solid materials.

To summarize, the theoretical approach using invariant theory has been intensively developed in recent years. It still has some challenges as far as coupled invariants are concerned, but the corresponding works are on the way. We believe that this approach will become more important in the next years because it has a clear physical basis that can be conveniently transferred into computational code.

#### 3.2.3. Effective Medium Theory

Since MAEs can be considered as composite materials, it is natural to employ effective medium theory (EMT) or effective medium approximations to calculate their macroscopic properties from the known physical properties of the constitutive materials. This approach has been developed in the works of Snarskii et al. [125,126,127,128]. The initial MAE microstructure was assumed to be random heterogeneous, in particular, randomly located spherical inclusions of the first phase (carbonyl iron) dispersed in a continuous polymer matrix (second phase). Contrary to the conventional EMT, the microstructure (i.e., mutual arrangement of filler particles) of composite material changes under the influence of an external field in the situation when dimensions and the shape of the sample remain constant. The following physical model was proposed: When the particle concentration φ is less than the critical threshold value $\varphi_{c}$, there is an assortment of finite clusters in the composite material (called a pre-cluster), which, with an increase in the concentration of inclusions φ→$\varphi_{c}$, will connect parts of the pre-cluster and form an infinite cluster. When a magnetic field is applied to an MAE specimen and the elastic matrix is compliant, the particles with the attached matrix can move within the specimen (until the elastic force from the matrix stops them) and thereby increase the relative number of particles (i.e., their concentration) in the pre-cluster. The main idea of the proposed theoretical description of effective properties in the case of the field-induced rearrangement of inclusions was that the increase in their concentration in the pre-cluster can be interpreted as a decrease in the percolation threshold $\varphi_{c}$. This means that the reduction of the difference ($\varphi_{c}$ − φ) is not attributed to a (local) increase in φ, but to a decrease in $\varphi_{c}$. With such a description, the percolation threshold $\varphi_{c}$ is no longer a constant, but is a function of the magnetic field that decreases with increasing ⟨H⟩: $\varphi_{c}=\varphi_{c}\left(\left\langle H\right\rangle \right)$, where ⟨…⟩=1/V∫…dV, V is the averaging volume, wherein the characteristic dimensions of the averaging region should be much larger than the correlation length *ξ*. The following dependence of the percolation threshold on the external magnetic field, as introduced by Mitsumata et al. [129], was used:

(15) $$\varphi_{c}\left(\left|\left\langle H\right\rangle \right|\right)=\varphi_{c0}exp\left(-\left|\left\langle H\right\rangle \right|/H_{c}\right),$$

where $H_{c}$ ischaracteristic magnetic field strength and $\varphi_{c0}$ is percolation threshold in the absence of a magnetic field. Comparisons with experiments showed that the $H_{c}$ has the order of magnitude 10^5^–10^6^ kA/m. To describe the magnetodielectric effect in MAEs, the authors of [125] used the modified Bruggeman–Landauer (BL) [130,131] approximation:

(16) $$\frac{\frac{\varepsilon^{e}-\varepsilon_{1}}{\varepsilon_{1}+2\varepsilon^{e}}}{\left[1+c\left(\varphi ,\varphi_{c}\right)\frac{\varepsilon^{e}-\varepsilon_{1}}{\varepsilon_{1}+2\varepsilon^{e}}\right]}\varphi +\frac{\frac{\varepsilon^{e}-\varepsilon_{2}}{\varepsilon_{2}+2\varepsilon^{e}}}{\left[1+c\left(\varphi ,\varphi_{c}\right)\frac{\varepsilon^{e}-\varepsilon_{2}}{\varepsilon_{2}+2\varepsilon^{e}}\right]}\left(1-\varphi \right)=0,$$

where $\varepsilon^{e}$ is the effective relative permittivity and $\varepsilon_{1}$, $\varepsilon_{2}$ are the relative permittivities of the consituitive materials. The addends in the denominators (emphasized by square brackets) proportional to $c\left(\varphi ,\varphi_{c}\right)$ represent the generalization of the BL approximation by Sarychev and Vinogradov (SV) [132]. According to [132], this renormalization is related to the additional contribution to the local field by the inclusions. Specifically, the SV term is:

(17) $$c\left(\varphi ,\varphi_{c}\right)=\left(1-3\varphi_{c}\right){\left(\frac{\varphi }{\varphi_{c}}\right)}^{\varphi_{c}}{\left(\frac{1-\varphi }{1-\varphi_{c}}\right)}^{1-\varphi_{c}},$$

In a later paper [126], this method has been generalized to describe the anisotropy of the magnetically induced changes of the effective permittivity. A reasonable agreement between theory and experiment was observed.

To describe the magnetorheological effect, the modified system of the classical self-consistent EMT for elasticity problem [133,134] was proposed:

(18) $$\left\{\begin{array}{c} \frac{\Omega_{1}}{1+s\left(\varphi ,\varphi_{c}\right)\Omega_{1}}\varphi +\frac{\Omega_{2}}{1+s\left(\varphi ,\varphi_{c}\right)\Omega_{2}}\left(1-\varphi \right)=0\\ \frac{\Theta_{1}}{1+s\left(\varphi ,\varphi_{c}\right)\Theta_{1}}\varphi +\frac{\Theta_{2}}{1+s\left(\varphi ,\varphi_{c}\right)\Theta_{2}}\left(1-\varphi \right)=0 \end{array}\right.,$$

Here the following notations were used:

$$\Omega_{i}=\frac{\frac{G_{i}}{G_{e}}\cdot \frac{1+\nu_{i}}{1+\nu_{e}}\cdot \frac{1-2\nu_{e}}{1-2\nu_{i}}-1}{1+\alpha_{e}\left(\frac{G_{i}}{G_{e}}\cdot \frac{1+\nu_{i}}{1+\nu_{e}}\cdot \frac{1-2\nu_{e}}{1-2\nu_{i}}-1\right)},\;\Theta_{i}=\frac{\frac{G_{i}}{G_{e}}-1}{1+\beta_{e}\left(\frac{G_{i}}{G_{e}}-1\right)},\;\alpha_{e}=\frac{1}{3}\cdot \frac{1+\nu_{e}}{1-\nu_{e}},\;\beta_{e}=\frac{2}{15}\cdot \frac{4-5\nu_{e}}{1-\nu_{e}},$$

and $G_{e}$*,* $\nu_{e}$ denote the effective shear modulus and the Poisson’s ratio, respectively, while $G_{1}$, $G_{2}$, $\nu_{1}$, $\nu_{2}$ are the values of these moduli in the first and second phases.

The new term $s\left(\varphi ,\varphi_{c}\right)$ is defined in the following way:

(19) $$s\left(\varphi ,\varphi_{c}\right)=\left(1-2\varphi_{c}\right){\left(\frac{\varphi }{\varphi_{c}}\right)}^{\varphi_{c}}{\left(\frac{1-\varphi }{1-\varphi_{c}}\right)}^{1-\varphi_{c}},$$

where $\varphi_{c}$ is the function of the external magnetic field (15) [127].

The concept of the field-dependent percolation threshold allowed one to describe in a unified manner the magnetodielectric effect, the non-monotonous field dependence of the magnetic permeability in MAEs [125] and to explain the order of magnitude for the giant or colossal MR effect. Therefore, the restructuring of the filler has to be taken into account when considering the MR effect in MAEs. Recently, Chougale et al. [135] pointed out that the hydrodynamic reinforcement factor *k* plays a key role in the drastic increase in the MR effect due to restructuring of the filler and argued that the divergence of *k* is equivalent to the definition of percolation threshold by Snarskii et al.

Interestingly, the model of movable percolation threshold predicts a significant change in a Poisson’s ratio of compliant MAEs in external magnetic fields. It was proposed to use the measurement of a Poisson’s ratio as a verification test for this theoretical model. The model does not exclude alternative mechanisms, which may be present simultaneously and should also contribute to the field-stiffening or magnetorheological effects (by further enhancement).

If a percolating structure comes into play in a composite material, its existence must be observed in several physical properties (cross-property relations) [128]. An important next step should be theoretical explanation of the empirical relationship (15) proposed in [129]. In particular, the relation of the critical magnetic field $H_{c}$ to the physical properties of a composite material and its constitutive components has to be established [128].

To summarize, the EMT is capable of offering a unified theoretical approach for description and explanation of different physical phenomena in MAEs, caused by the restructuring of particles. Describing the field-induced anisotropy of mechanical properties of MAEs is currently a challenge because a suitable formulation of self-consistent EMT for elasticity problem is not available.

The overall challenges that lie before the field of MAE continuum modeling stem from the very foundations of this approach. The equations describing the material behavior are much more complex than classic equations of particle motion; those equations are frequently nonlinear and include coupled magnetomechanical material relations. Various aspects of such models require further refining: taking into account the viscoelastic properties of the polymer matrices, rigorously incorporating nonlinear magnetization models and magnetic anisotropy into constitutive equations, analyzing the coupling terms of the material energy function in more detail to better capture the behavior of soft MAEs in the presence of strong magnetic fields. As discussed in Section 3.2, several attempts to tackle these problems have been made recently, however a more complete continuous model of MAE behavior that combines all of the mentioned factors has not been developed yet. On the other hand, it should be mentioned that considerable progress has been made in regard to formalization of fundamentals of MAE physics. Several works that provide a deep analysis of thermodynamics of magneto-dielectric-mechanical systems as well as the general properties of the corresponding energy functions have emerged. One can expect that more rigorous and general solutions of the magnetomechanical continuum problems that do not rely on the small deformations and weak magnetic field approximations will appear in scientific literature in the coming years.

### 3.3. Rheological Modeling

Rheological modeling is the simplest approach when it comes to description of MAE behavior and it is intrinsically tied to experimental studies. The dynamic material behavior is described phenomenologically on a macroscopic scale using a system of mechanical elements connected in series or in parallel. This approach is similar to describing the behavior of an electronic circuit with a circuit diagram. Each mechanical element in the equivalent circuit has its own stress-strain relation that can depend on the external magnetic field, and the resulting stress-strain relation of the equivalent circuit corresponds to material behavior. Model parameters are calculated via error functional minimization by comparing the modeling output with the data of dynamic mechanical loading experiments for MAEs in the presence of external magnetic field. Typical elements include linear elastic springs, Newtonian dashpots, plastic elements, friction elements, nonlinear springs and fractional viscoelastic elements. If trends of the model parameters’ dependences on the material composition and magnetic field are established and approximated, the model can have predictive value.

Dynamic mechanical analysis is a phenomenological approach, so any rheological model is adjusted to describe specific objects and experiments and load cases. The parameters of the model elements in this approach are obtained via fitting the dependences of characteristics of a typical sample on the external stimuli obtained as a result of experimental measurements. Multicomponent composition, the presence of particle–matrix interfaces, magnetic and elastic memory effects as well as magnetomechanical coupling lead to a very complex transient rheological response of MAEs. It was shown that in temporally stepwise changing magnetic fields and oscillation amplitudes, at least three exponential functions are required to reasonably describe the time behavior of the storage shear modulus of MAEs on long time scales exceeding tenths of minutes [136]. The corresponding time constants of three identified structuring processes differ by one order of magnitude [136]. The presence of different relaxation mechanisms at different time and length scales requires the construction of rheological schemes with several relaxation and retardation times. Attempts to use classical rheological schemes to describe the viscoelastic behavior of magnetoactive elastomers lead to significant complication of the model [137], which is especially noticeable when it is required to describe the behavior of the material for a wide range of magnetic field strength values. The viscoelastic behavior of magnetoactive elastomers can change significantly with a change in the magnetic field, therefore a sufficiently flexible rheological model is required. The discussed approach was also implemented for MAEs using non-classical rheological elements [137,138,139,140,141], however, the dependence of the model parameters on the external field makes constructing rheological models without a priori knowledge of the magnetic properties of the material rather challenging. The information about MAE magnetization curves must then be obtained using some other experimental or theoretical research methods. In particular, in [142] the magnetization model of MAEs was combined with magnetic dipole theory and quantitative description of frequency-dependent shear modulus in various magnetic fields, as a result, the authors proposed a generalized Maxwell model connected in parallel with a magneto-induced modulus model which was able to describe frequency and magnetic field dependencies of the dynamic shear modulus of isotropic MAEs based on silicone rubber filled with carbonyl iron microparticles. The parameters of the magnetization model were obtained from fitting experimental magnetization curves.

There exists a different class of rheological elements intrinsically suitable for viscoelasticity modeling by virtue of the mathematical definition of their stress-strain relation and possessing sufficient flexibility to describe a wide variety of processes without complicating the rheological model. This class of elements is called fractional rheological elements and, as the name suggests, the stress-strain relation for such elements is represented with a fractional order differential equation. Fractional elements exhibit memory effects and not only generalize rheological models, but also fundamentally enrich them. There exist several forms of fractional differential operators, one of them, the left-handed Riemann–Liouville fractional derivative of order α (0 < α < 1) which was used, for instance, in [143,144], can be expressed as follows:

(20) Dxα0RLfx=1Γ1−αddx∫0xfξdξx−ξα,

Here $\Gamma \left(x\right)$ is Euler’s gamma function. The stress-strain relation for this fractional element has the following form: σ=c⋅Dtα0RLε=c⋅εα. Thus, it is characterized by two parameters-the fractional order α and the viscoelasticity coefficient c, which makes it more flexible than the classical ones. For the limiting cases α→0 and α→1, one has the following relations connecting fractional viscoelasticity with classical viscoelasticity:

(21) limα→0DxαaRLfx=fx, limα→1DxαaRLfx=ddxfx,

It means that the fractional element becomes a Hookean spring in the limit of α = 0, and it turns into a Newtonian dashpot in the opposite limit α = 1.

**Figure 10:**
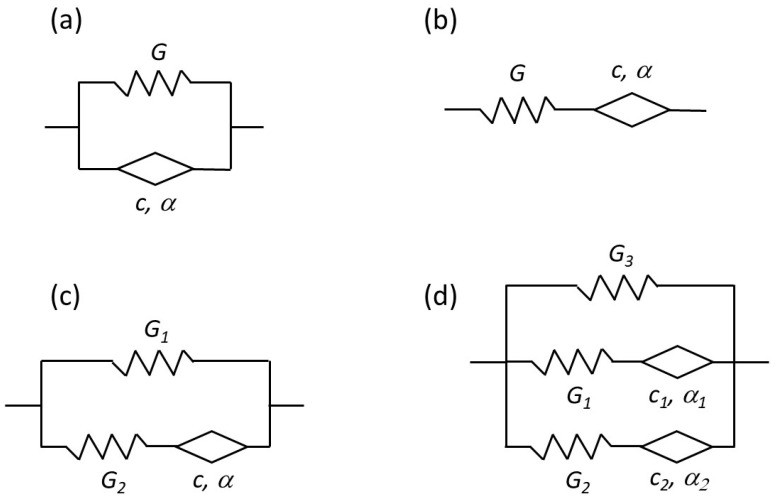
Fractional rheological models: (**a**) Kelvin–Voigt model; (**b**) Maxwell model; (**c**) Zener model; (**d**) generalized Maxwell model with two branches. G, $G_{1}$, $G_{2}$ are the shear moduli of corresponding springs; c, $c_{1}$, $c_{2}$ are viscoelasticity coefficients of the fractional elements; α, $\alpha_{1}$, $\alpha_{2}$ are the order parameters of the fractional elements.

In recent years, works describing the MAE behavior using fractional rheological models have emerged. It has been shown that even the simplest classical models, namely, the Maxwell and the Kelvin–Voigt models of a viscoelastic medium in principle can describe the viscoelastic behavior of a MAE sample if the classical dashpot is replaced by the fractional element (Figure 10a,b). It has been shown that these simplest one-fractional-element rheological schemes can adequately describe the viscoelasticity of MAEs with various concentrations of magnetic filler in small magnetic fields where the major contribution to the dynamic response comes from a polymer network [143]. They can also be used in saturating magnetic fields where the dynamic response is dominated by the response of a strong magnetic network formed by the filler particles [143]. In intermediate magnetic fields, when the magnetomechanical coupling plays an important role and restructuring of magnetic particles takes place one needs to use more sophisticated fractional schemes. The works [144,145,146,147] used the Zener model that corresponds to the standard linear solid model (Figure 10c). In [147], the four-parameter Zener model with a fractional element instead of a classical dashpot was used to describe the stress relaxation behavior of anisotropic MAEs. The model was in good agreement with experimental data obtained in both single- and multi-step relaxation tests. In [146], the Zener model was modified not only by replacing the dashpot with the fractional element but also using springs with field-dependent spring stiffness. It can describe well the dynamic mechanical behavior of isotropic MAEs in various magnetic fields both in time and frequency domains. In [143], a more accurate model was proposed; it corresponds to the generalized Maxwell model with two branches (Figure 10d). Fitting the experimental data with the results from the generalized Maxwell model has demonstrated that the evolution of the fractional order parameters for this model follows the three scenarios of the MAE viscoelastic properties changes observed in small, intermediate and strong magnetic fields and it was interpreted in terms of MAE microstructure evolution. The authors of Ref. [148] compared the performance of two branches: classical Maxwell model with six fitting parameters and a fractional Maxwell model with five fitting parameters and concluded that the latter is better in terms of accuracy, simplicity and flexibility. The works [149,150] proposed combined phenomenological models consisting of a fractional rheological circuit that describes viscoelasticity, as well as elements corresponding to other aspects of material behavior. In [149], elements describing the friction and dipole–dipole interaction of filler particles were considered. In [150], elements that reproduce the nonlinear magnetization of the material, nonlinear elasticity, and elastoplasticity were used. In [151], the fractional Maxwell model was coupled with a stochastic linearized Bouc–Wen component. This eight-parameter model described viscoelastic as well as the magnetic field and strain dependent behavior of MAEs with a high accuracy exceeding 91%. In [152], the elastic–plastic model with linear hardening was adopted.

To summarize, rheological modeling is actively developing nowadays. The main trend is to combine rheological schemes with models of field-dependent and MAE-composition-dependent dynamic shear modulus and to enhance the predictive capacity of the rheological models in a wide range of loads and magnetic field strengths. In spite of a lack of physical meaning in this type of approach, the rheological models can be very effective in modeling dynamic response of MAE elements used in various practical applications, in particular, for vibration control and isolation. The prospect of combining rheological modeling with continuum and/or microscopic models seems very promising. It could allow one to create models that are reasonably easy to use and have clearly defined physical meaning inherent to the two other approaches mentioned in this article. Rheological modeling is also being used in MAE hysteresis models, which is another promising field of MAE research that would be better discussed in a separate review. Rheological models found currently in the literature do not describe anisotropic response of MAEs and utilize simple approximations for the dependences of the rheological elements’ properties on the applied magnetic field. Thus, it follows that such flaws should be addressed in order to create rheological models with higher degrees of generality.

## 4. Multi-Scale Modeling Approaches

**Figure 11:**
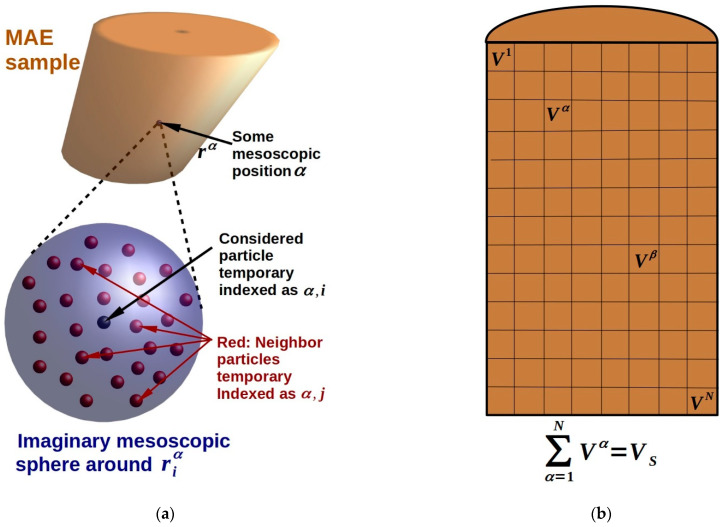
(**a**) Sketch of the decomposition of an MAE sample into short- and long-range effects; (**b**) Formal discretization of sample volume $V_{s}$ into mesoscopic portions $V_{\alpha }$, $\alpha \in \left[1,N\right]$. On such scales any particle microstructure appears a homogeneous continuous distribution [153].

The most widely spread and the most physically consistent combined modeling framework found in literature is the combination of microscopic (or mesoscopic) and macroscopic scales. Both the microstructure and the sample shape effects are taken into account in combined modeling, leading to a more holistic material behavior description. Local characteristics are used to define macroscopic characteristics in these approaches. Naturally, solving multiscale problems is a complex task that requires a lot of theoretical considerations and computational resources. Direct characterization of the material as a composite medium is difficult, and an alternative to such an approach is the homogenization of the micro-scale medium, which creates combined micro-macroscopic models. Micro-scale models can be based on solving equations of motion for filler particles or treating them as a microcontinuum. The scale of the problem under study is of utmost importance for theoretical description of MAEs as not only are the mechanical and magnetic phenomena connected in such materials, but the processes occurring on the scales of filler particles and the entire sample are closely connected as well. Combined microstructural and macrostructural models (Figure 11) bridge the gap between the different scales using various homogenization procedures through constructing representative volume elements (RVEs) and averaging with volume integration. RVE characteristics are used to obtain macroscopic parameters that are then used in a sample-scale model. An important assumption employed in most multi-scale models is the length scales separation hypothesis according to which the different spatial scales in the material are geometrically decoupled, so any microscopic or mesoscopic structural element is seen as a material point on a macroscopic scale. The processes occurring on different scales influence each other by iteratively transferring field variable data between scales (for example, average magnetization of a microscopic element is assigned to a single point in a macroscopic model). There are several approaches to microscopic element modeling, microscale homogenization, sample modeling and description of the surrounding volume.

### 4.1. Representative Element Homogenization Approaches

Multi-scale modeling is usually based on the internal microstructure of the material. Thus, the homogenization procedures are based on calculations of macroscopic sample behavior using averages of microscopic quantities in representative elements. The representative elements can be obtained using periodic boundary conditions, ordered filler structure models or statistical procedures that calculate the element size based on the deviation of the resulting element characteristics from the macroscopic average.

A framework for analytical magnetoelastic homogenization in MAEs was given and discussed in [154] for a static two-dimensional case. The approach was based on microscopic volume averaging and partial decoupling of the variational magnetomechanical problem. Uniaxial loading in the presence of external magnetic field for a sample containing elliptic particles of various sizes was studied in [155] in a quasi-static regime using analytical considerations in tandem with FEM modeling. This variational approach was further developed by Danas in [156], where it was described as periodic homogenization. The local homogenization problem was substituted with a simpler periodic filler structure that consisted of single particle cells, and the variational problem was changed correspondingly to account for perturbations arising due to the employed periodic approximation. The influence of filler volume concentration, distribution, particle shape and orientation on the magnetization and magnetostriction of two-dimensional MAEs was studied. Recently, numerical homogenization was carried out in [157] for the case of isotropic three-dimensional MAEs with magnetically hard filler. The periodic homogenization procedure was improved to include RVEs with several particles of varying sizes and evolution in time (incremental periodic homogenization). The proposed model also included magnetic dissipation potential. An explicit analytical model based on invariant theory was also developed and compared with homogenization results. The analytical model was verified by solving the MAE cantilever beam deflection problem. It was found that for moderately stiff and stiff polymer matrices (with shear modulus higher than 150 kPa) the model’s predictions were in line with the homogenization results as well as experimental data found in literature.

In [78], a procedure for constructing a representative volume element of a MAE for the case of spherical filler particles was presented. The authors compared two approaches to averaging and homogenization of the properties of a medium. The first approach involved deriving the weak form of the continuum equations of the medium using the variational representation and a numerical solution of the resulting equations. The second approach was based on generating random distributions of filler particles as a part of an iterative process with each iteration checking if the current element is fit to be a representative volume element using a statistical procedure. The calculation of the parameters of a real representative element was then performed on the basis of the convergence of the physical characteristics of the system (elasticity modulus and effective magnetic permeability) with an increase in the number of statistical realizations. The calculation of these characteristics was carried out using the finite element method. The authors calculated the dimensions of a representative volume element for the case of a two-dimensional system, a polymer matrix with strain energy density in the neo-Hookean form, as well as a fixed filler concentration, and specific mechanical and magnetic properties of system objects. It was shown that the results obtained using both of the considered approaches to the RVE modeling largely coincided.

A microstructure-based constitutive model for hard-magnetic MAEs was considered in [158]. Under free-stress conditions of the post-cured MAEs, the composite was assumed to reach an equilibrium state where the polymeric network balances the dipole–dipole interactions of magnetic particles. Within a single framework, the model described the overall magneto-mechanical response of the MAEs with magnetically hard filler considering the specific contributions of its phases. The numerical results revealed that a pre-deformation of the polymeric network is required to reach consistent mechanical balance in the presence of magnetized particles. The change in the distances between particles during the MAE deformation led to changes in the dipole–dipole interactions affecting the overall response of the composite. This effect was noted to be particularly important in the absence of an external magnetic field.

In [159], the hard-magnetic, compliant MAE was modeled as a three-dimensional micropolar continuum body, which was subjected to external magnetic stimuli. From the angular momentum balance law, it was deduced that the Cauchy stress tensor in these materials cannot be symmetric. Therefore, the micropolar continuum theory [160], with inherently asymmetric stress tensor, was chosen as a rational candidate for modeling the deformation of these materials. In micropolar continuum theory each material particle is associated with a microstructure that can undergo only rigid rotations independently from the surrounding medium. Therefore, each particle contains six degrees of freedom: three translational which are assigned to the macro-element, and three rotational ones which are related to the micro-structure. From the kinetic point of view, the interaction between two adjacent surface elements was considered via a couple vector in addition to the traditional traction vector, which led to the definition of couple stress tensor. It was shown that the presented formulation can successfully predict the deformation of hard magnetic soft materials under various loading and boundary conditions.

### 4.2. FE2-Approach

FE2 method is a robust multi-scale FEM modeling approach that assumes the existence of two classic continuum scales: microscopic and macroscopic. Each macroscopic node of the FE mesh corresponds to a microscopic element of the material. In the case of so-called weakly coupled scales, this microscopic element is a representative volume element (RVE) of the sample. For the sake of simplicity, weakly coupled scales models are more widely used than their strongly coupled counterparts that do not assume any kind of statistical representation of the material microstructure. Another important assumption of the FE2 method is the separation of scales: the scale of an RVE is much smaller than the basic scale of the macroscopic continuum.

In order to describe the material behavior, BVPs on both scales must be solved. This is an iterative process with each iteration consisting of several steps. First, the values of macroscopic variables (such as stress, strain and magnetic field) are assigned to each node starting from the initial material state. These values are then used as boundary conditions for the microscopic BVP for an RVE solved using the weak formulation. The obtained results are then averaged via a homogenization procedure and the averaged values are assigned to the nodes of the macroscopic mesh. Finally, the macroscopic BVP is solved, and the next iteration is prepared. This process is carried out until the changes between iterations become negligible. MAE is a nonlinear material and because of that obtaining a solution is a demanding process in terms of the computational resources it requires. Various linearization methods can be employed to decrease the resource requirements.

The work [161] presented a framework for solving magnetomechanical BVPs using FEM on microscopic and macroscopic scales for large strains. Homogenization theory was applied to the BVP on microscale to create a self-consistent model where macroscopic quantities (deformation gradient and *H*-field) were used to obtain the average stress and *B*-field in microscopic elements by employing the generalized Hill–Mandel condition [162,163] in a microscopic BVP:

(22) $$\bar{P}:\delta \bar{F}-\bar{\vec{B}}\cdot \delta \bar{\vec{H}}={\left\langle P:\delta F\right\rangle }_{V}-{\left\langle \vec{B}\cdot \delta \vec{H}\right\rangle }_{V},$$

where the line above a symbol is used to denote a macroscopic physical quantity, δ is the variation or increment of a physical quantity and $\;{\left\langle *\right\rangle }_{V}$ denotes the volume average of ∗. This condition provides connection between macroscopic quantities and microscopic averages. These averages were then used in a linearized macroscopic BVP to calculate new deformation gradient and *H*-field. Microscopic elements containing a single filler particle were considered with varying microstructure orientation that translates into varying rotation angles for the microscopic cells. Material stiffening in uniform magnetic field under shear load was studied, and the obtained results were noted to be in accordance with experimentally observed phenomena. Magneto-electric-mechanical coupling was considered in [164]. The effects of the sample shape were additionally studied in [165]: two-dimensional rectangular and elliptic samples were considered in order to compare the results with analytical predictions. Continuum theory based around the influence of Maxwell stress on the sample boundary shape was used to obtain analytical estimations. The effects of the magnetic properties and the shape of the sample on Maxwell tractions on its boundary were discussed. It was shown that information about internal stress state of the sample can be obtained using the tractions measured on its boundary.

Fourier transform method can be used in order to reduce the computational complexity of the microscopic problem. In [166] a comprehensive step-by-step algorithm of Fast Fourier Transform method for nonlinear magnetoelasticity was provided along with its mathematical justifications. It was used to simulate the material response to external load and magnetic field in 2D and 3D for neo-Hookean polymer medium and hyperbolic magnetization model. This work provided a rigorous framework that allows one to incorporate Fourier space-based homogenization into multiscale MAE modeling.

### 4.3. Mean Field Approach

Another way of taking into account both microscopic and macroscopic effects was proposed by Ivaneyko et al. [167], which they refer to as the mean field theory. It is obvious that even when using the dipole approximation for magnetic interactions, calculating the contributions to the magnetic field and energy from every single filler particle in an MAE sample is an incredibly demanding task. In the dipole theory, the magnetic field inside the sample can be expressed as a superposition of dipolar contributions from every particle at a given point in space. A dimensionless shape factor *f* can then be introduced:

(23) $$\begin{array}{cc} f=\frac{1}{4\pi \varphi }\underset{i\neq j}{\sum }\frac{3{\left(r_{ij}\right)}_{m}^{2}-r_{ij}^{2}}{r_{ij}^{5}} & {\left(r_{ij}\right)}_{m}=r_{ij}\left({\vec{r}}_{ij},{\vec{e}}_{m}\right){\vec{e}}_{r} \end{array},$$

where φ is the volume concentration of the ferromagnetic filler, ${\vec{r}}_{ij}=r_{ij}{\vec{e}}_{r}$ is the vector connecting the centers of particles with numbers *i* and *j*, ${\vec{e}}_{m}$ is the vector denoting the direction of the magnetic moment of particle *j*. The shape factor represents the distribution of filler particles in the sample.

Another important aspect of the modeling approach in question is the decomposition of the problem into two parts: a mesoscopic sphere surrounding the chosen point in the material and the rest of the sample. Due to the distance dependence of the dipolar magnetic field, the particles located far enough from a given particle can be considered to be independent from it. The mesoscopic part of the magnetic field heavily depends on the microstructure while the sample (or macroscopic) part of the magnetic field depends on the sample shape and average magnetization:

(24) $$f=f_{micro}+f_{macro},$$

where $f_{micro}$ is the sum over particles inside the mesoscopic sphere and $f_{macro}$ is the sum over particles outside of it. The macroscopic sum can be replaced by an integral due to the fact that differences between contributions from different particles become negligible far away from the center of the mesoscopic sphere. The macro part of the function f can be calculated more easily due to further homogenization of the field and density variables, and it is related to the demagnetization factor of the sample. The micro part’s complexity depends on the local distribution of filler particles. This approximation was dubbed by the authors the dipolar mean field theory. Bulk magnetization can then be obtained using a chosen magnetization model for the filler particles and will depend on f. In this work equilibrium deformation and magnetization for simple cubic, body centered cubic, hexagonal close-packed and tetragonal lattices representing filler particle distributions are calculated using the simplest form of elastic and magnetic energy of the sample. The results of the direct summation in f are compared to the results obtained within the approximation framework, and the agreement was found to be good.

This theoretical approach to describing the properties of magnetoactive elastomers was further developed in [168,169,170]. The magnetic field inside the material can be represented as a combination of a local field, which is determined by the mutual arrangement of particles in the region near the selected particle, and a macroscopic field, which is determined by the average characteristics of the sample and its form factor. The filler particles were described as linearly magnetizable magnetic dipoles, and the total magnetization was calculated for an ellipsoid-shaped MAE sample with a magnetic filler concentration not exceeding 20% by volume. Using the linear theory of elasticity, the total energy of an ellipsoidal sample of a magnetically active elastomer was also calculated. The result of both of these assumptions was an integral equation for the magnetization, which was solved iteratively. The short-range effects were ignored. Sample magnetization and deformation were obtained for random filler particle distributions and cylindrical structures. In [168] the dipolar mean field approach was compared with full field FEM simulations, and it was concluded that for systems with sufficiently low filler concentrations both approaches provide qualitatively and quantitatively the same results. In [169,170] using the basic principle that dictates that filler particles tend to form elongated structures inside the polymer matrix in the presence of a magnetic field, and within the framework of this approach an assumption was made that the filler structures could be modeled as magnetized continuous medium areas within the sample volume. With that assumption in effect, magnetic field inside the material can be calculated as an integral of the dipolar contributions from each particle using a density function instead of a direct sum of those contributions. This can be equated to ensemble averaging over all possible configurations of the microstructure. Additionally, the contribution from the particle located at a given point must be excluded, so the integration area is truncated. This assumption allows the model to take into account the microstructural effects arising from dipole–dipole interparticle interactions while at the same time building a continuous model that is more appropriate for analytical studies and easier to solve numerically. In [169], within the framework of this approach, the influence of the initial distribution of filler particles on the energy of a magnetoactive elastomer was taken into account under the assumption of physically small local deformations of the material and weak magnetic fields. It was shown that the initial distribution of particles affects the mechanical behavior of the composite, in particular, the type of material magnetostriction: compression or stretching. A modification of such a model using the mean field theory was proposed in [170].

In [171], another interpretation of the mean field approach was offered: magnetization was considered to be a superposition of the average filler magnetization and a local perturbation. The theoretical framework was generalized by introducing operator formalism. The nonlinear magnetization models were also considered. The work [153] further generalized this approach by discretizing the sample volume into a set of mesoscopic volumes with the microstructure in each of them not directly affecting the other mesoscopic volumes (akin to FE2 approach). The sample was characterized by a macroscopic average magnetization, each mesoscopic volume—by a mesoscopic average magnetization that deviated from the macroscopic average, and each point inside a mesoscopic volume—by a local deviation from mesoscopic average. Each mesoscopic volume can also be modeled as a dipole with characteristics corresponding to the averages of the local fields, and thus $f_{macro}$ is analogous to $f_{micro}$, but represents the dipolar structure on a different scale without taking deviations into consideration. The Taylor linearization of the general non-linear magnetization with respect to the deviation of the local magnetic field from the average field served as the basis for obtaining self-consistent magnetization equation. This work presented a way to decouple and explicitly calculate the leading magnetic effects corresponding to different scales of the MAE internal structure.

In [172], several theoretical models were used to describe the MAE behavior and, in particular, the influence of microstructural effects on the magnetic field-induced deformations of MAEs. The macroscopic behavior was described using invariant theory and free energy-based constitutive equations. The microscopic/mesoscopic effects were modeled using two different approaches: micro-continuum modeling with invariant theory as well as FEM simulations and dipolar particle interaction theory together with matrix-mediated two-body and three-body interaction theory proposed by [173]. The results obtained using two microscopic approaches were compared for 3D helical filler chains, and a good agreement was demonstrated for interparticle distances corresponding to the applicability limits of magnetic dipole theory. This work aimed to establish a computationally efficient (compared to full-field micro-scale FEM models) algorithm for calculating local MAE response to external magnetic fields within a multiscale modeling framework on the basis of dipolar mean field theory and classic linear theory of elasticity for media containing hard inclusions.

**Figure 12:**
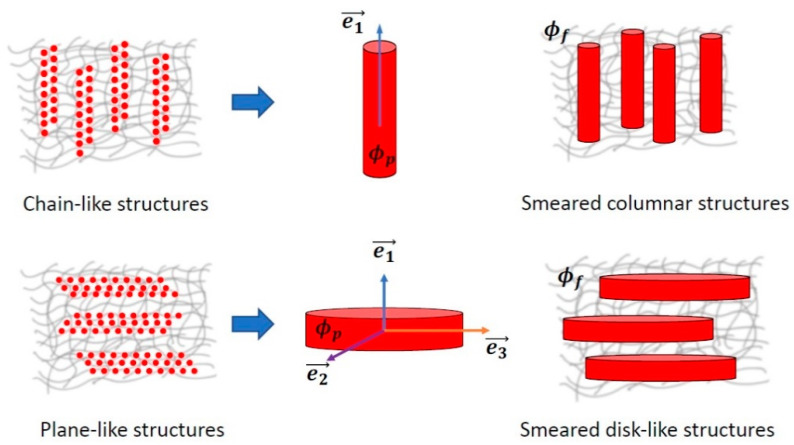
Smearing of magnetic particles (with total volume fraction φ) into columnar and disk-like structures. $\varphi_{p}$ is the volume fraction of magnetizable particles inside a smeared structure, and $\varphi_{f}=\varphi /\varphi_{p}$ represents the volume fraction of smeared structures inside an elastomer matrix. MAEs, in both cases, exhibit transverse isotropy along a unit vector ${\vec{e}}_{1}$ [135].

The work [135] further developed the approach proposed in [170]; namely, chain-like and plane-like structures of the filler were modeled as continuous rods and discs, respectively (Figure 12). MAE sample deformation and its elastic modulus were then described using invariant theory for transverse isotropic materials to derive the mechanical part of the free energy and dipolar mean field theory with a smeared filler particle density function to derive the magnetic part of the free energy.

It follows that the current promising trend is the opportunity to move away from describing ferromagnetic filler microstructures as uniform lattices towards more complex distributions that resemble the real material structure. Using regular lattices in theoretical descriptions of MAEs leads to undesirable artifacts appearing in the obtained material behavior, so introducing new analytical approaches to approximating filler clusters with simpler shapes or otherwise reducing their structural complexity is one of the requirements for more universal and less computationally intense multiscale modeling of MAEs.

To summarize, multi-scale theoretical approaches seem to be the most promising line of research because they are capable of solving very complex problems while keeping the advantages of simplified theoretical description at a single scale. The inherent nonlinearity of the problems obtained within coupled multi-scale frameworks seems to constitute the main challenge for the practical implementations of such methods, which leads to high computational costs. Various multi-scale approaches are currently being actively developed and improved in order to understand the connection between microstructural changes and material sample response to external magnetic fields and mechanical loads. Multi-scale modeling shows the importance of both the filler structure inside an MAE sample and the sample shape, as well as the interplay between these factors for both understanding fundamentals of MAE behavior and achieving desired performance of MAE-based devices in practical applications. The most challenging aspect of the multi-scale approaches discussed in this section is their analytical and computational complexity. The work on creating and utilizing less computationally demanding algorithms for multi-scale MAE modeling would greatly benefit the scientific community and would naturally accelerate progress in obtaining a comprehensive and reasonably complete model of magnetopolymer composite materials.

## 5. Conclusions and Outlook

The above considerations clearly demonstrate that a significant progress has been achieved in theoretical modeling of MAEs in the past five to ten years. Prior to that, the research was mostly focused on experiments revealing new physical effects and experimental elucidation of dependences of them on the material composition and excitation conditions. The advances in the understanding of the underlying physical phenomena in MAEs have been made in all theoretical approaches described above.

What should the future directions of theoretical research on MAEs be? In our opinion, the answer is determined by the observable trends in the experimental works on MAEs and their potential applications. The current technology of MAE fabrication allows for synthesis of materials with more sophisticated compositions. The filler becomes more complex, with particles of different physical natures (e.g., soft-magnetic, hard-magnetic, and non-magnetic inclusions), different particle sizes (e.g., nm, sub-µm, and µm-sized particles) and shapes (e.g., spherical, rod-like and plate-like particles). Modern methods of additive manufacturing also allow us to fabricate elastomers with specific (sophisticated) particle distributions (e.g., ordered, randomly or nonuniformly distributed filling particles) in different spatial regions of a MAE-based functional element. This is done to achieve the desired response of a functional element (e.g., actuator). The resulting compounds may even include a composite material inside another composite material (e.g., ferrofluid in an MAE) or several polymers combined to form a matrix. Future theoretical works will face the challenge of a necessity to describe the physical effects in magnetoactive polymers with increasing complexity of composition not only qualitatively but also quantitatively. To do this, the nonlinear effects of both the mechanical and magnetic properties of constitutive materials have to be treated without the linearized approximations. The theoretical research of MAEs has not yet reached its goals. On the contrary, the subject of this review paper will be flourishing in the coming years, once theoretical modelling is a practical tool for designing MAE materials for functional applications.

## Data Availability

Not applicable.

## Abbreviations

The following abbreviations are used in this manuscript:

BL	Bruggeman-Landauer
BVP	Boundary value problem
DPD	Dissipative particle dynamics
EMT	Effective medium theory
FE	Ferroelectric
FEM	Finite element method
FM	Ferromagnetic
FORC	First order reversal curve
MAE	Magnetoactive elastomer
MD	Molecular dynamics
HM	Hard magnetic
MNG	Magnetic nanogel
MR	Magnetorheological
SM	Soft magnetic
RVE	Representative volume element
SV	Sarychev and Vinogradov
